# Altered Microbiomes in Bovine Digital Dermatitis Lesions, and the Gut as a Pathogen Reservoir

**DOI:** 10.1371/journal.pone.0120504

**Published:** 2015-03-17

**Authors:** Martin Zinicola, Fabio Lima, Svetlana Lima, Vinicius Machado, Marilia Gomez, Dörte Döpfer, Charles Guard, Rodrigo Bicalho

**Affiliations:** 1 Department of Population Medicine and Diagnostic Sciences, Cornell University, Ithaca, New York, United States of America; 2 Department of Medical Sciences, School of Veterinary Medicine, University of Wisconsin, Madison, Wisconsin, United States of America; University of Alberta, CANADA

## Abstract

Bovine digital dermatitis (DD) is the most important infectious disease associated with lameness in cattle worldwide. Since the disease was first described in 1974, a series of *Treponema* species concurrent with other microbes have been identified in DD lesions, suggesting a polymicrobial etiology. However, the pathogenesis of DD and the source of the causative microbes remain unclear. Here we characterized the microbiomes of healthy skin and skin lesions in dairy cows affected with different stages of DD and investigated the gut microbiome as a potential reservoir for microbes associated with this disease. Discriminant analysis revealed that the microbiomes of healthy skin, active DD lesions (ulcerative and chronic ulcerative) and inactive DD lesions (healing and chronic proliferative) are completely distinct. *Treponema denticola*, *Treponema maltophilum*, *Treponema medium*, *Treponema putidum*, *Treponema phagedenis* and *Treponema paraluiscuniculi* were all found to be present in greater relative abundance in active DD lesions when compared with healthy skin and inactive DD lesions, and these same *Treponema* species were nearly ubiquitously present in rumen and fecal microbiomes. The relative abundance of *Candidatus Amoebophilus asiaticus*, a bacterium not previously reported in DD lesions, was increased in both active and inactive lesions when compared with healthy skin. In conclusion, our data support the concept that DD is a polymicrobial disease, with active DD lesions having a markedly distinct microbiome dominated by *T*. *denticola*, *T*. *maltophilum*, *T*. *medium*, *T*. *putidum*, *T*. *phagedenis* and *T*. *paraluiscuniculi*. Furthermore, these *Treponema* species are nearly ubiquitously found in rumen and fecal microbiomes, suggesting that the gut is an important reservoir of microbes involved in DD pathogenesis. Additionally, the bacterium *Candidatus Amoebophilus asiaticus* was highly abundant in active and inactive DD lesions.

## Introduction

Bovine digital dermatitis (DD) is the most relevant infectious disease associated with lameness in cattle worldwide [1]. Cows diagnosed with DD have reduced milk production, impaired reproductive performance, and increased risk of culling [2],[3],[4], with an estimated cost per case of US$133 [5]. The reported prevalence of DD ranges from 21.2% to 29.2% [6],[7] and the 2007 USDA National Animal Health Monitoring System survey reported that 61.8% of lameness cases in bred heifers and 49.1% in adult cows are caused by DD [8]. Therefore, if we assume a DD incidence rate of 25%, then in the United States (~9 million dairy cows) and the European Union (~24.5 million dairy cows) combined, the annual economic loss from DD exceeds US$1.1 billion. Animal welfare is a further burden of DD in addition to the estimated economic losses.

DD in cattle was first described in Italy [9], and during the last 40 years multiple studies have investigated potential DD etiological agents, identifying consistently an overwhelming presence of several spirochetes from the genus *Treponema* in DD lesions [10], [11],[12],[13],[14],[15]. The three most common spirochete types found associated with DD lesions are *Treponema denticola-like*, *Treponema medium*, and *Treponema phagedenis-like* [12],[16],[17]. It has been shown that *Treponema phagedenis-like* can cause immunosuppression of bovine macrophages and undermine not only the innate immune response, but wound repair as well, which may explain the progression and persistence of DD lesions [18]. The use of fluorescent *in situ* hybridization (FISH) analyses revealed that *Treponema* spp. are seen mostly in the deeper parts of DD lesions, near the interface with healthy tissue [11],[19]. Cattle with DD develop high levels of antibodies against *Treponema* spp. soon after infection occurs; however, these antibodies do not offer protection against the development of lesions [20],[21],[22]. Our research group previously investigated the microbiomes of different strata of DD lesions, revealing the existence of 166 predominant phylotypes [14]. *Treponema* spp. were the most prominent group detected in DD deep biopsies, but they were absent in healthy skin samples [14]. Recently, Krull et al. (2014) [15] used shotgun and 16S rRNA metagenomic sequencing to investigate the microbial diversity across different stages of DD using a novel scoring system based on lesion morphology and associated microbiome detect particularly the start of the clinical signs. Fungi and viruses were not present in the lesions, and *Treponema* spp. predominated in the advanced lesions but had relatively low abundances in the early stages of the lesions [15]. Additionally, the consortium of *Treponema* spp. identified at the onset of the disease changed considerably as the lesions progressed through the designated morphologic stages [15].

Although it seems very likely that *Treponema* spp. play a critical role in the pathogenesis of DD, attempts to induce the disease by skin inoculation with pure cultures of these bacteria were largely unsuccessful [23]. Other bacteria, including *Fusobacterium necrophorum*, *Porphyromonas* spp., *Bacteroides* spp., *Campylobacter* spp., *Guggenheimella* spp., *Borrelia* spp., and *Dichelobacter nodosus* have also been identified in DD lesions, suggesting a polymicrobial etiology and possible synergistic relationship among *Treponema* spp. and other microbes [15],[19],[24],[25],[26].

The infection reservoirs and transmission routes of DD remains unclear. A few reports investigated the bovine gastrointestinal tract using molecular techniques and it was concluded that bovine DD treponemes do not appear to form part of the normal gut microbiota [17],[27]. However, recent work by Klitgaard et al. (2014), using high-throughput sequencing, identified DD-associated treponemes in environmental samples (e.g., manure slurry) collected from dairy farms [28].

Notwithstanding of decades of research, the pathogenesis of DD remains controversial and under study. Thus, full characterization of the microbiomes of both deep and superficial strata in different stages of DD-lesion progression, as well as investigation of the gut microbiome and the environment as a potential source of the pathogens in DD lesions, are necessary.

Here we characterized the microbiomes of deep and superficial strata of healthy skin and DD lesions in dairy cows affected by different stages of DD by sequencing the 16S rRNA gene using the Illumina MiSeq platform. Additionally, we investigated the gut microbiome using shotgun and 16S metagenomic techniques to determine its potential role as a DD pathogen reservoir. Our results show that the microbiomes of healthy skin, active DD lesions (ulcerative and chronic ulcerative), and inactive DD lesions (healing and chronic—non- ulcerative) are completely distinct. *Treponema denticola*, *Treponema maltophilum*, *Treponema medium*, *Treponema putidum*, *Treponema phagedenis* and *Treponema paraluiscuniculi* had greater relative abundance in active DD lesions when compared with both healthy skin and inactive DD lesions, and these *Treponema* species were also nearly ubiquitously present in rumen and fecal microbiomes. *Candidatus Amoebophilus asiaticus*, a novel bacterium for DD samples, had increased relative abundance in both active and inactive lesions when compared with healthy skin. Collectively, our results reveal that ulcerative lesions of DD have a particular microbiome dominated by *T*. *denticola*, *T*. *maltophilum*, *T*. *medium*, *T*. *putidum*, *T*. *phagedenis* and *T*. *paraluiscuniculi*, and the gut microbiome hosts this consortium of *Treponema* spp. associated with DD pathogenesis.

## Materials and Methods

### Ethics statement

This study was carried out in strict accordance with the recommendations of The Animal Welfare Act of 1966 (AWA) (P.L. 89–544) and its amendments 1970 (P.L. 91–579); 1976 (P.L. 94–279), 1985 (P.L. 99–198) regulate the transportation, purchase, care, and treatment of animals used in research. The research protocol was reviewed and approved by the Institutional Animal Care and use Committee of Cornell University (Protocol number: 2008–0096). The skin biopsy sample collections from cattle affected with DD were authorized by the farm owner, who was aware of the procedure.

### Healthy skin and digital dermatitis lesion sample collection

**Fig 1:**
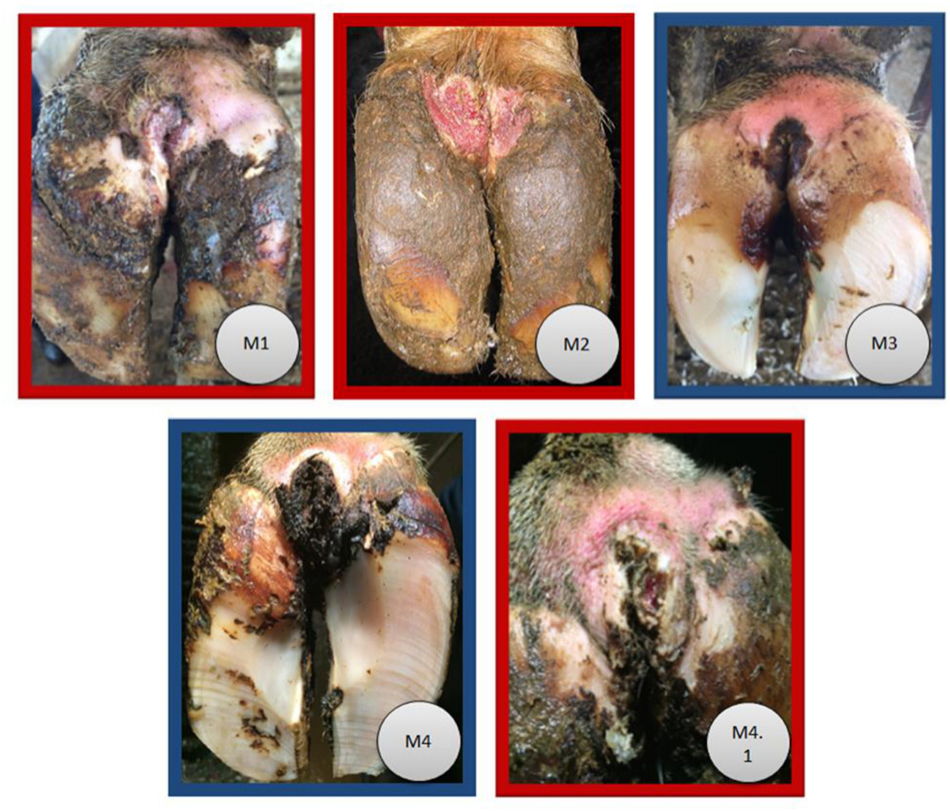
Digital dermatitis lesion scoring system modified from Döpfer et al. (1997) and Berry et al. (2012) [25],[29]. M1 is an early-stage ulcerative lesion (0–2 cm diameter); M2 is an ulcerative painful lesion with a diameter >2 cm; M3 is the healing stage with a lesion covered by a scab; M4 is the chronic stage characterized by dyskeratosis or surface proliferation; and M4.1 consists of a chronic lesion with a small area of ulceration. The highlight colors on the border of each image cluster the digital dermatitis lesions into two different types: active (red border) and inactive (blue border).

Samples from healthy skin and DD lesions were collected from Holstein dairy cows housed in three different dairy farms located near Ithaca, NY. Lesion classification was based on the scoring method described by Döpfer et al. (1997) [25] and modified by Berry et al. (2012) [29] and depicted in Fig. 1. Briefly, M1 is an early-stage ulcerative lesion (0–2 cm diameter) that is not painful on palpation; M2 is the classical ulcerative stage with a diameter >2 cm that is often painful on palpation; M3 is the healing stage with a lesion covered by a scab; M4 is the chronic stage characterized by dyskeratosis or surface proliferation that is generally not painful; and M4.1 describes a chronic lesion with an small area of ulceration. A total of 140 samples were collected from 89 dairy cows, 89 from DD lesions (M1, n = 1; M2, n = 30; M3, n = 5; M4, n = 26; M4.1, n = 27) and 51 from healthy skin (M1, n = 1; M2, n = 16; M3, n = 5; M4, n = 15; M4.1, n = 14), which were harvested using a 0.6-cm diameter punch biopsy instrument (Biopsy Punch, Miltex Inc., PA). We did not have sufficient M1 lesions to draw conclusions about their microbiome though. The punch biopsy was performed in the center of the DD lesion. A representative healthy skin samples were collected from the same hoof affected by DD, 2 cm from the center of the DD lesions, which were cleaned with water and gently dried with a paper towel before the samples were harvested. Collected samples were placed in a sterile 2.0 ml microcentrifuge tube and transported on ice to the laboratory. Samples generally included the epidermis, dermis and hypodermis. In the laboratory, under sterile condition, the samples were trimmed with a #22 scalpel blade to recover two different layers, the first 2 mm (superficial) and the rest of the sample (deep), resulting in 280 samples, which were stored in sterile 2.0 ml microcentrifuge tubes at -20°C until analysis.

### Fecal and rumen sample collection

Fecal (14 cows) and rumen (8 cows) samples were randomly collected from lactating Holstein cows housed on the same three commercial dairy farms where healthy skin and DD lesions biopsy samples were collected. DD history and the presence of DD lesion at samples collection were not recorded. Fecal samples were collected via the rectum using clean rectal sleeves. After collection, fecal samples were placed in 50-ml sterile conical tubes and kept on ice until transported to the laboratory in Ithaca, NY, where the samples were preserved at -80°C.

We sampled the rumen using a non-invasive procedure with the aid of a scientifically evaluated and commercially available oro-ruminal sampling device (Flora Rumen Scoop, profs-product, Guelph, Canada) [30]. After sample collection, 50-ml aliquots of rumen fluid were stored in sterile conical tubes and kept on ice until transported to the laboratory in Ithaca, NY, where the samples were preserved at -80°C.

### DNA extraction

Healthy skin samples and DD lesions were washed 3 times with nuclease-free water after being trimmed, and incubated at 56°C for 12 h with 40 μl of proteinase K (IBI Scientific), 180 μl of tissue lysis buffer, and 40 μl of lysozyme (QIAamp DNA Minikit, Qiagen, Valencia, CA, USA) to maximize bacterial DNA extraction. 250 mg of post-incubation heathy skin samples, DD lesions, and fecal samples were placed in PowerBead Tubes (PowerSoil DNA Isolation kit, MO BIO Laboratories, Inc., Carlsbad, CA, USA), and settled in a Mini-Beadbeater-8 (Biospec Products, Battersville, OK, USA) for microbial cell disruption. DNA extraction was performed using a PowerSoil DNA Isolation Kit (MO BIO Laboratory Inc.) following the manufacturer’s recommendation. Rumen fluid samples were thawed and subsequently homogenized by vortexing for 3 min. 1-ml aliquots of each rumen fluid sample were centrifuged at 13,200 rpm (16,100 rcf) in an Eppendorf 5415R centrifuge for 10 min at room temperature. The supernatant was discarded and the remaining pellet was resuspended in 400 μl of nuclease-free water. Isolation of genomic DNA was then performed by using a QIAamp DNA minikit (Qiagen) according to the manufacturer’s instructions, except that 400 mg of lysozyme was added to the bacterial suspension and incubated for 12 h at 56°C to maximize bacterial DNA extraction. DNA concentration and purity were evaluated by optical density using a NanoDrop ND-1000 spectrophotometer (NanoDrop Technologies, Rockland, DE, USA) at wavelengths of 230, 260 and 280 nm.

### PCR amplification of the V4 hypervariable region of bacterial 16S rRNA genes

The 16S rRNA gene was amplified by PCR from individual metagenomic DNA samples of healthy skin, DD lesions and feces using barcoded primers. For amplification of the V4 hypervariable region of the bacterial 16S rRNA gene, primers 515F and 806R were used according to a previously described method [31] optimized for the Illumina MiSeq platform. The earth microbiome project (http://www.earthmicrobiome.org/) [32] was used to select 140 different 12-bp error-correcting Golay barcodes for the 16S rRNA PCR, as previously described [31]. The 5′-barcoded amplicons were generated in triplicate using 12–300 ng DNA template, 1× GoTaq Green Master Mix (Promega, Madison, WI), 1 mM MgCl_2_, and 10 μM of each primer. The PCR conditions for the 16S rRNA gene consisted of an initial denaturing step of 94°C for 3 min, followed by 35 cycles of 94°C for 45 s, 50°C for 1 min, and 72°C for 90 s, and a final elongation step of 72°C for 10 min. Replicate amplicons were pooled and purified with a QIAquick PCR Purification Kit (Qiagen, Valencia, CA, USA), and visualized by electrophoresis through 1.2% (wt/vol) agarose gels stained with 0.5 mg/ml ethidium bromide before sequencing. Blank controls, in which no DNA was added to the reaction, were performed. Purified amplicon DNA was quantified using the Quant-iT™ PicoGreen dsDNA Assay Kit (Life Technologies Corporation, Carlsbad, CA, USA).

### Shotgun sequencing

An aliquot of all rumen DNA samples was normalized to 0.2 ng/μl to be used as input to the XT DNA Sample Prep Kit (Illumina Inc. San Diego, CA, USA). Tagmentation of samples was done using 1 ng of template, as directed by the manufacturer. Following tagmentation, PCR amplification was done according to the manufacturer’s instructions using a unique combination of indexing primers for each of the 8 samples to allow for multiplexing of samples. Following amplification, short DNA fragments were removed from each library by using AMPure XP bead purification, and then normalized with Library Normalization beads/additives. In preparation for cluster generation and sequencing, equal volumes of normalized library were combined, diluted in hybridization buffer and heat denatured, according to the Nextera XT protocol. Pair-end sequencing was performed using the MiSeq Reagent Kit v3 (500 cycle) on the Illumina MiSeq platform.

### Sequence and statistical analysis

Amplicon aliquots of healthy skin, DD lesions and feces were standardized to the same concentration and then pooled into 3 different runs (two runs with 140 samples for healthy skin and DD lesions, and a third with 14 samples for feces) according to individual barcode primers for the 16S rRNA gene. Final equimolar libraries were sequenced using the MiSeq reagent kit v2 (300 cycles) on the MiSeq platform (Illumina, Inc., San Diego, CA, USA). The obtained 16S rRNA gene sequences were processed through the open source software pipeline Quantitative Insights Into Microbial Ecology (QIIME) version 1.7.0-dev [33]. Sequences were filtered for quality using established guidelines [34]. Sequences were binned into Operational Taxonomic Units (OTUs) based on 97% identity using UCLUST [35] against the Greengenes reference database [36], May 2013 release. Low-abundance clusters were filtered and chimeric sequences were removed using USEARCH [35]. Eleven samples with less than 1000 sequences from healthy skin and DD lesions were excluded, leaving 269 samples for downstream analyses. The representative sequences for each OTU were compared against the Greengenes database for taxonomy assignment, and only full-length, high-quality reads (-r = 0) were used for analysis. Additionally, we generated a species-level OTU table using the MiSeq Reporter Metagenomics Workflow. The MiSeq Reporter classification is based on the Greengenes database (http://greengenes.lbl.gov/) and the output of this workflow is a classification of reads at multiple taxonomic levels: kingdom, phylum, class, order, family, genus, and species. For shotgun sequencing of rumen samples, raw data files were de-multiplexed and converted to. fastq using Casava v.1.8.2 (Illumina, Inc, San Diego, CA, USA). Fastq files were concatenated and uploaded to the MG-RAST [37] server for analysis to determine relative abundance for phylum, order, class, family, genus and species, using a non-redundant multi-source protein annotation database (M5NR), a maximum e-value of 1.10–5, minimum identity of 60% and minimum alignment length of 15 bp.

Dependent variables were the mean relative abundance of microbial types and independent variables were the different types skin lesions. Variables with a *P*-value < 0.05 were deemed statistically different.

Using the obtained OTU information, we evaluated each sample’s richness using the Chao1 index, which is a nonparametric estimator of the minimum richness (number of OTUs) and is based on the number of rare OTUs (singletons and doublets) within a sample. The Chao1 index means (± SD) for healthy skin samples, active DD lesions and inactive DD lesions were then compared using a general linear model within JMP Pro 11 (SAS Institute Inc., NC).

The relative abundance of microbial taxon types in superficial and deep samples of healthy skin, inactive DD lesions and active DD stages, and for each of the stages recorded (M2, M3, M4 and M4.1) were compared using general linear models (ANOVA) on JPM Pro 11 (SAS Institute Inc., NC).

The relative abundance of different bacterial taxa in each sample were used as covariates in stepwise discriminant analysis models built in JMP Pro 11. Variables were removed in a stepwise manner until only variables with a *P* value < 0.001 were retained in the final model. A series of multivariable screening analyses using JMP Pro 11 was performed to determine which bacterial species were most important to differentiate the healthy skin microbiome from the microbiomes of active DD lesions and inactive DD lesions. We used false discovery rate (FDR) to correct for multiple comparisons in our screening analysis. Given the large sample size of DD data and the equally large number of hypotheses tested, we used a very strict probability of FDR (FDR-probability ≤ 0.001) to minimize type-1 statistical errors. Linear correlation matrixes (Pearson correlation coefficient) were generated to illustrate the level of correlation of the bacterial taxa selected by the screening model as the major microbes differentiating active DD lesions from inactive DD lesions and healthy skin samples.

Fastq data obtained as results of sequencing skin samples of cows diagnosed with digital dermatitis lesions and feces of lactating dairy cows were uploaded to the sequence read archive (SRA) on National Center for Biotechnology Information (NCBI) web page tool (http://www.ncbi.nlm.nih.gov/sra) to make the files available for a public databases; accession number SRR1562236. The rumen fluid shotgun sequencing data have been deposited in the MG-RAST (http://metagenomics.anl.gov/metagenomics.cgi?page=Home) database and are publically available under the following accession numbers (4565633.3, 4565634.3, 4565635.3, 4565636.3, 4565637.3, 4565638.3, 4565639.3, and 4565640.3).

## Results

### Sequencing results, core microbiome description, and genera relative abundance

Quality-filtered reads for the 16S rRNA sequences for skin samples were demultiplexed, yielding 21,345,584 sequences (median = 73,934; range = 3,134–337,766) in total with a median length of 301 bases per read, and an average coverage of 79,352 sequences per sample. The average number of operational taxonomic units (OTUs) per sample was 50,810. Fecal samples generated 1,717,191 sequences (median = 123,948; range = 105,904–159,024) and rumen samples 2,783,926 (median = 270,011; range = 45,949–815,543).

**Fig 2:**
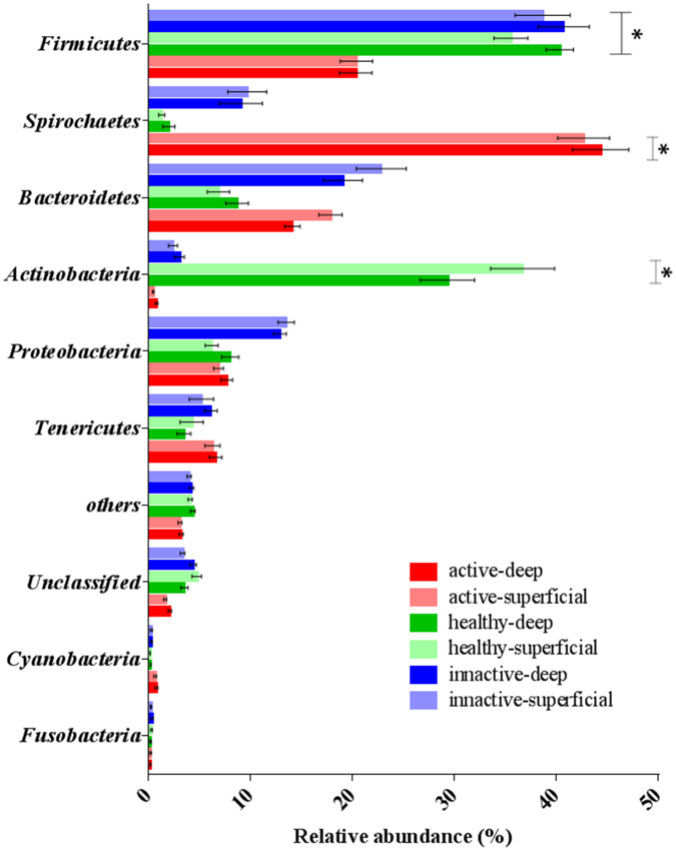
Relative abundance of phyla from superficial (light colors) and deep (dark colors) samples of healthy skin (green), active (red) and inactive (blue) digital dermatitis lesions. Error bars represent standard error of the mean. The asterisks indicate significant differences between healthy skin and active or inactive digital dermatitis lesions. *P < 0.05.

Comparison of relative abundance at the phylum level revealed the microbiomes of healthy and DD lesions to be dominated by eight major phyla: Firmicutes, Spirochaetae, Bacteroidetes, Actinobacteria, Proteobacteria, Tenericutes, Cyanobacteria and Fusobacteria (Fig. 2). No differences in relative abundance between superficial and deep samples were identified at the phylum level (Fig. 2). The relative abundance of the phylum Firmicutes was reduced for active DD lesions (*M* = 20.3%, *SEM* = 1.59) when compared with healthy skin (*M* = 37.9%, *SEM* = 1.50) and inactive DD lesions (*M* = 39.6%, *SEM* = 2.60) (Fig. 2). On the other hand, the phylum Spirochaetae was markedly increased in active DD lesions (*M* = 43.5%, *SEM* = 2.65) when compared with healthy skin (*M* = 1.6%, *SEM* = 0.44) and inactive DD lesions (*M* = 9.3%, *SEM* = 2.01) (Fig. 2). The relative abundance of the phylum Actinobacteria was increased in healthy skin (*M* = 33.0%, *SEM* = 2.90) when compared with active DD lesions (*M* = 0.6%, *SEM* = 0.10) and inactive DD lesions (*M* = 2.7%, *SEM* = 0.45) (Fig. 2). No differences among the other phyla evaluated or between deep and superficial samples were detected (Fig. 2).

### Relative abundance of the most prevalent bacteria in deep and superficial samples in healthy skin, active DD lesions, and inactive DD lesions

**Fig 3:**
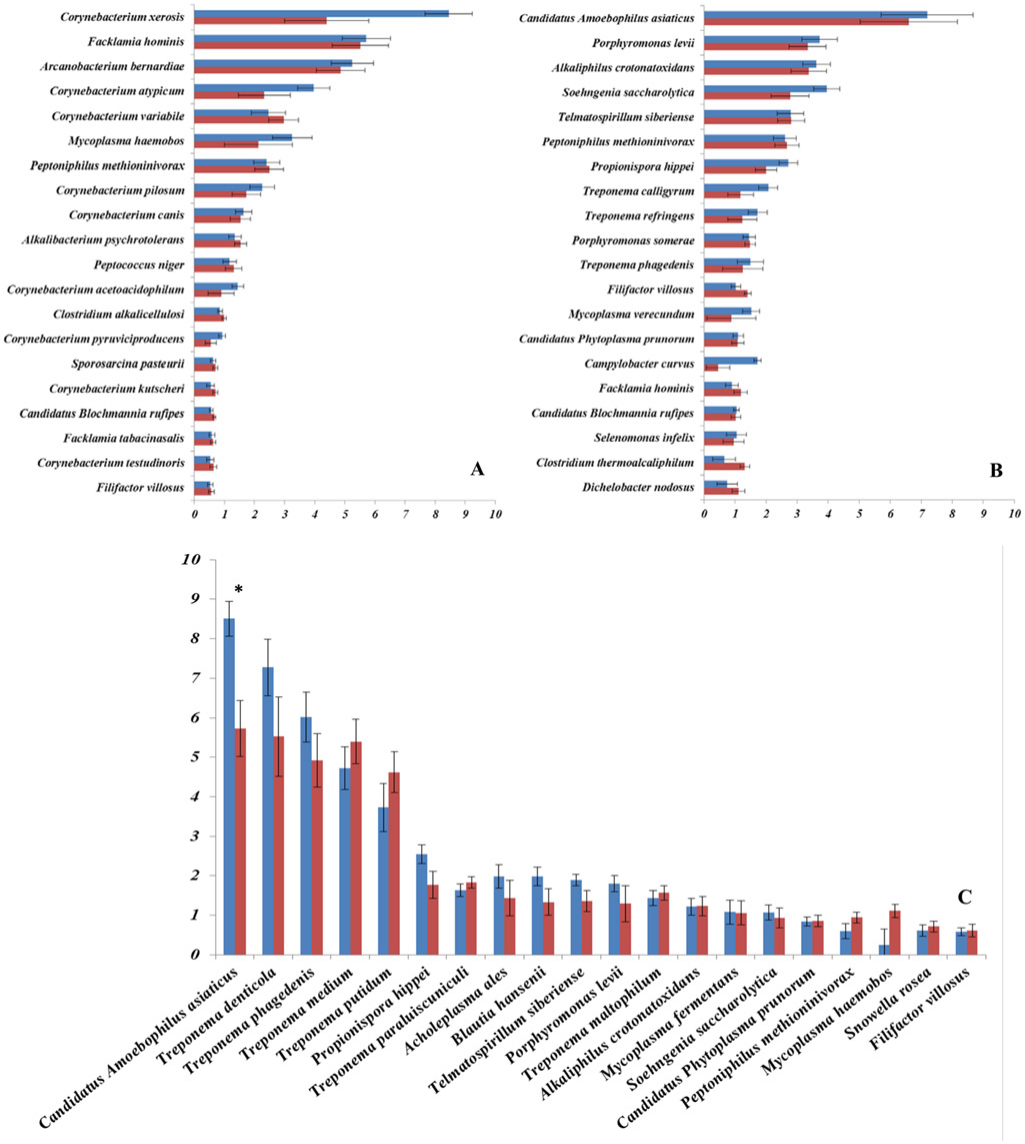
Average relative abundance of the 20 most common bacteria detected in deep (red bars) and superficial (blue bars) samples of healthy skin (A), inactive digital dermatitis lesions (B) and active digital dermatitis lesions (C). Error bars represent the standard error of the mean. The asterisk indicates a significant difference between superficial and deep samples. * = *P* < 0.05

The relative abundance of the 20 most prevalent bacteria found in the microbiomes of the superficial and deep samples of healthy skin, active DD lesions and inactive DD lesions are depicted in Figs. 3A, 3B and 3C, respectively. No differences (*P* > 0.05) for the 20 most prevalent bacteria were found between the superficial and deep samples in healthy skin (Fig. 3A) and in inactive DD lesions (Fig. 3B). However, for active DD lesions, the relative abundance of the bacterium *Candidatus Amoebophilus asiaticus* was increased (*P* < 0.05) in superficial samples when compared with deep samples (Fig. 3C).

### Discriminant analysis and Chao1 richness index

**Fig 4:**
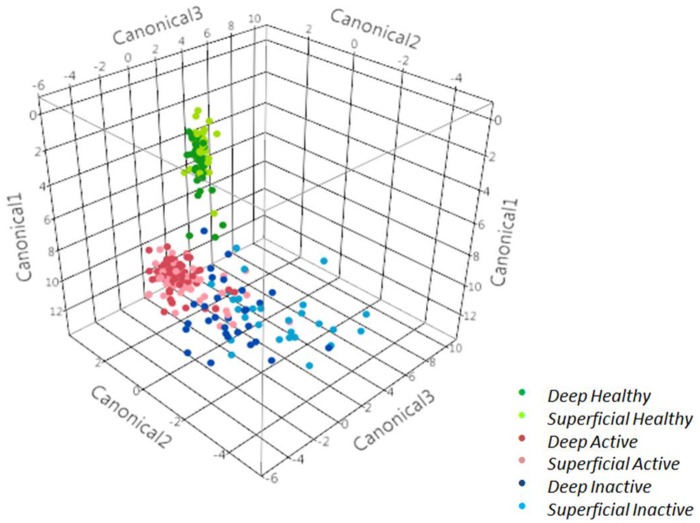
Discriminant analysis of superficial and deep strata samples from healthy skin, inactive digital dermatitis lesions and active digital dermatitis lesions.

Healthy skin samples were strongly discriminated from active and inactive DD lesions, and considerable discrimination between active and inactive lesions was also indicated by stepwise discriminant analysis (Fig. 4). Differences in the microbial diversity of superficial and deep strata samples between healthy skin and DD lesions (active and inactive) are illustrated by Canonical 1 (Fig. 4). Differences between active and inactive lesions are illustrated by Canonical 2 (Fig. 4). Canonical 3 illustrates differences, mostly minor, between superficial and deep samples of inactive lesions (Fig. 4). The canonical scores for each bacterial taxon that were used to discriminate the microbiomes of healthy skin, active DD lesions, and inactive lesions of superficial and deep samples are presented in S1, S2 and S3 Figs., respectively.

Similar discriminatory results were observed when samples were classified according to DD lesion stages (healthy, M1, M2, M3, M4 and M4.1) (S4 Fig.). Differences between healthy skin samples and lesion stages M1, M2 and M4.1 (active lesions), as well as M3 and M4 (inactive lesions) are mostly illustrated by Canonical 1 (S4 Fig.). Canonical 2 illustrates the differences between M3 lesion scores and M1, M2 and M4.1 lesion scores (S4 Fig.). Differences between lesion stage M4 and lesion stages M1, M2 and M4.1 are illustrated by Canonical 3 (S4 Fig.). The bacterial species scores for canonical scores 1, 2 and 3 are reported in S1 Table.

**Fig 5:**
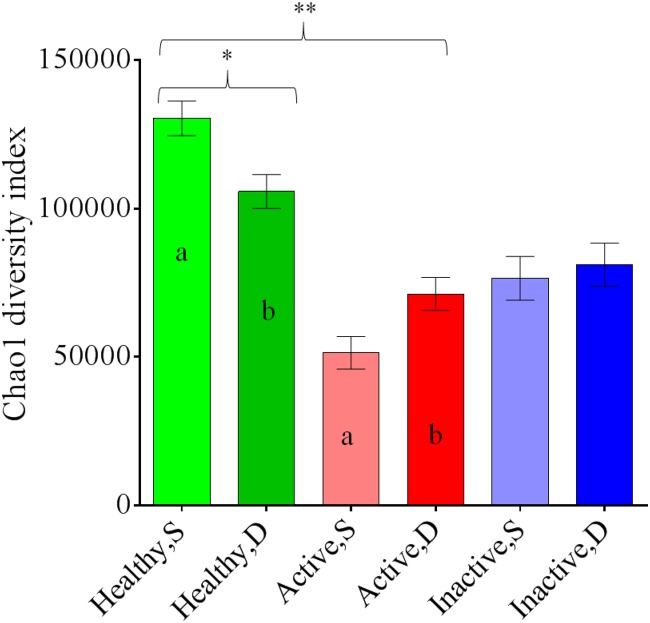
Bar graph illustrating the mean Chao1 index for superficial (S) and deep (D) strata samples of healthy skin, active digital dermatitis lesions, and inactive digital dermatitis lesions. Error bars represent standard errors. One asterisk means a significant difference (*P < 0.01) between healthy skin and lesion types. Two asterisks mean a significant interaction (**P < 0.01) for sample and lesion types. Different letters (a, b) indicate significant differences between different strata within a sample type.

The Chao1 richness index was greater for healthy skin samples than for active DD lesions and inactive DD lesions (Fig. 5). An interaction between sample type (superficial and deep) and lesion type (healthy, active and inactive) was identified, with the deep stratum samples of healthy skin having a lower diversity as indicated by Chao1 index compared to the superficial stratum samples of healthy skin, and the deep stratum samples of active DD lesions having an increased Chao1 index compared to the superficial stratum samples of active lesions (Fig. 5).

### Screening analysis

**Fig 6:**
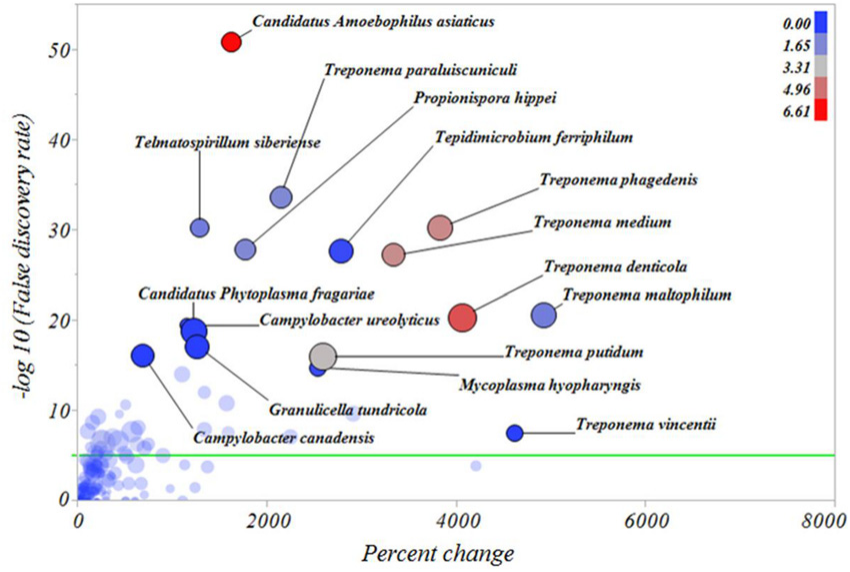
Percentage increase of bacterial types from healthy skin samples to active digital dermatitis lesions. The Y axis represents the robust LogWorth of the false discovery rate and the X axis represents the percentage increase in relative abundance when comparing healthy skin samples to active digital dermatitis lesions. The sizes of the circles represent the effect size and the colors represent the relative abundance of each individual bacterial type in active digital dermatitis lesions (color legend upper right corner). Green line represents *P* < 0.00005.

**Fig 7:**
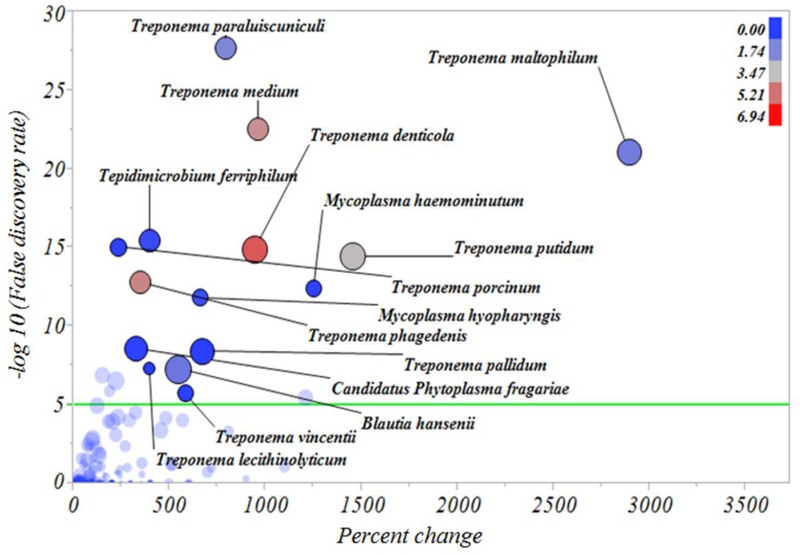
Percentage increase of bacterial types from inactive digital dermatitis lesions to active digital dermatitis lesions. The Y axis represents the robust LogWorth of the false discovery rate and the X axis represents the percentage increase in relative abundance when comparing inactive digital dermatitis lesions to active digital dermatitis lesions. The sizes of the circles represent the effect size and the colors represent the relative abundance of each individual bacterial type in active digital dermatitis lesions (color legend upper right corner). Green line represents *P* < 0.00005.

**Fig 8:**
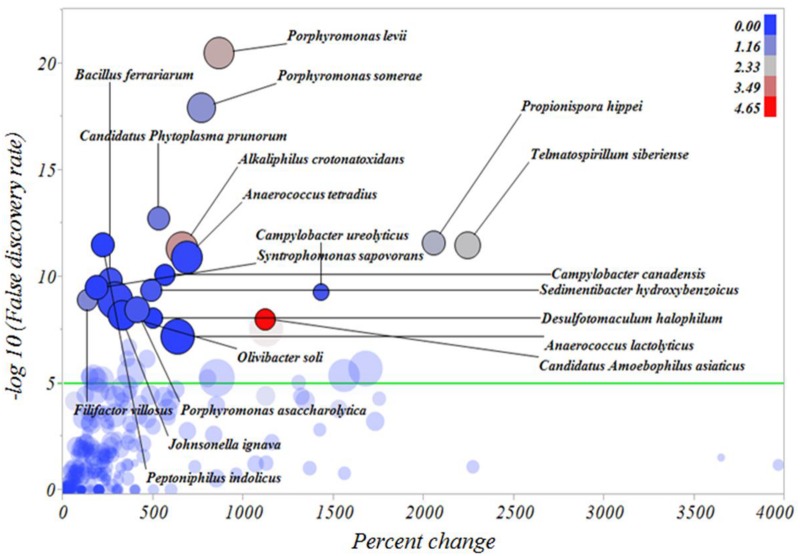
Percentage increase of bacterial types from healthy skin to inactive digital dermatitis lesions. The Y axis represents the robust LogWorth of the false discovery rate and the X axis represents the percentage increase in relative abundance when comparing healthy skin samples to inactive digital dermatitis lesions. The sizes of the circles represent the effect size and the colors represent the relative abundance of each individual bacterial type in inactive digital dermatitis lesions (color legend upper right corner). Green line represents *P* < 0.00005.

In an attempt to identify the major microbial types distinguishing the microbiomes of healthy skin, inactive DD and active DD lesions, a series of screening analyses was performed. The results of the screening analysis indicated a markedly increased relative abundance of a consortium of *Treponema* spp., specifically *T*. *denticola*, *T*. *maltophilum*, *T*. *medium*, *T*. *paraluiscuniculi*, *T*. *phagedenis*, and *T*. *putidum*, in active DD lesions compared with healthy skin (Fig. 6). Among all the microbes important for differentiating active DD lesions from healthy skin samples, the bacterium *Candidatus Amoebophilus asiaticus*, never reported previously in DD lesions, had the highest relative abundance (Fig. 6). Likewise, *T*. *denticola*, *T*. *maltophilum*, *T*. *medium*, *T*. *paraluiscuniculi*, *T*. *phagedenis* and *T*. *putidum* had increased relative abundance in active DD lesions when compared with inactive DD lesions (Fig. 7). The bacteria *Candidatus Amoebophilus asiaticus*, *Porphyromonas levii*, *Phorphyromonas somerae*, *Propionispora hippie*, and *Tematospirillum siberense* were increased in inactive DD lesions when compared with healthy skin samples (Fig. 8). The percentage reductions of the major microbial types in comparisons of active lesions versus healthy skin, inactive lesions versus healthy skin, and active lesions versus inactive lesions are given in S5, S6 and S7 Figs., respectively.

**Fig 9:**
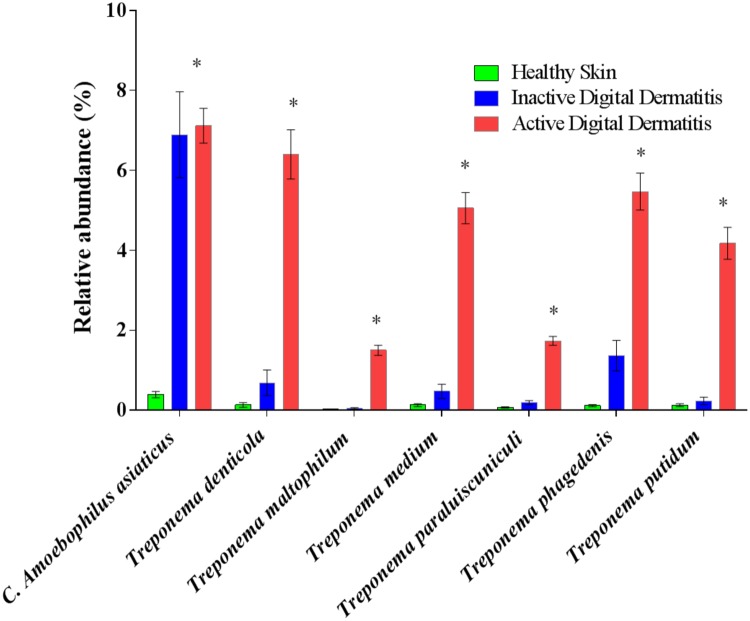
Relative abundance of the major bacterial species associated with digital dermatitis (DD) in healthy skin, inactive DD lesions and active DD lesions. Bacterial types were selected based on the top ranked robust LogWorth of the false discovery rate and average relative abundance of bacterial types in healthy skin, inactive DD lesions, and active DD lesions. Asterisks mean significance. *P < 0.05.

Microbial types highly associated with active DD lesions were selected based on robust LogWorth of the false discovery rate and relative abundance. The relative abundance of *T*. *denticola*, *T*. *maltophilum*, *T*. *medium*, *T*. *paraluiscuniculi*, *T*. *phagedenis* and *T*. *putidum* were markedly increased in active DD lesions when compared with healthy skin and inactive DD lesions (Fig. 9). Moreover, the proportion of *Candidatus Amoebophilus asiaticus* was increased in active and inactive DD lesions when compared with healthy skin samples (Fig. 9). Likewise, the consortium of *Treponema* spp. was increased in lesion stages M2 and M4.1 (active lesions) when compared with either lesion stages M3, M4 (inactive lesions) or healthy skin (S8 Fig.), and *Candidatus Amoebophilus asiaticus* was increased in lesion stages M2, M4 and M4.1 when compared with lesion stages M1, M3 or healthy skin samples (S8 Fig.).

### Linear correlation matrix

**Fig 10:**
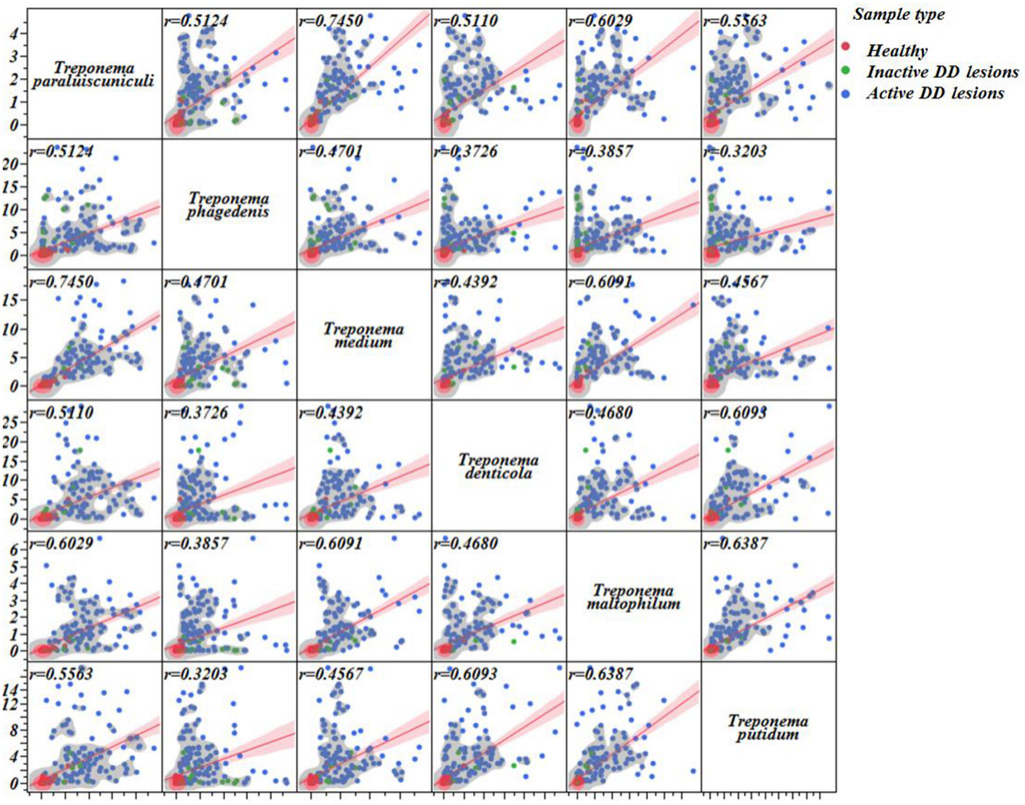
Linear correlation matrix illustrating the associations among all *Treponema* species that were highly associated with digital dermatitis. Linear correlation line (red line) and respective 95% C.I. (shaded red) as well as correlation coefficients for each association (upper left corner) are provided. A nonparametric density contour was used to illustrate how healthy (red dots), inactive (green dots), and active digital dermatitis (blue dots) samples are concentrated on each graph.

A linear correlation matrix analysis was performed to illustrate the correlation among all *Treponema* spp. that were found to be highly associated with active DD lesions (Fig. 10). Correlation coefficients above *r* = 0.32 were found for all bacteria evaluated. *T*. *paraluiscuniculi* was highly associated with *T*. *medium* (*r* = 0.74) and *T*. *maltophilum* (*r* = 0.60). *T*. *maltophilum* was also highly associated with *T*. *medium* (*r* = 0.60) and *T*. *putidum* (*r* = 0.63). *T*. *denticola* was highly associated with *T*. *putidum* (*r* = 0.60). *T*. *paraluiscuniculi* had correlation coefficients greater than *r* = 0.50 with the other *Treponema* spp. (Fig. 10).

### Relative abundance of Treponema species in fecal and rumen samples

**Fig 11:**
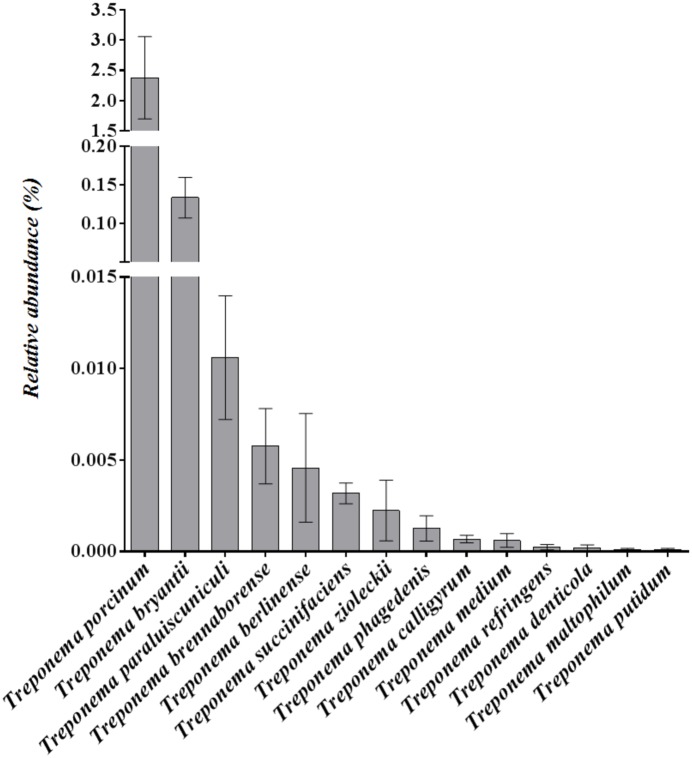
Relative abundance of *Treponema* species in fecal samples from 14 lactating dairy cows. Error bars represent standard error of the mean.

**Fig 12:**
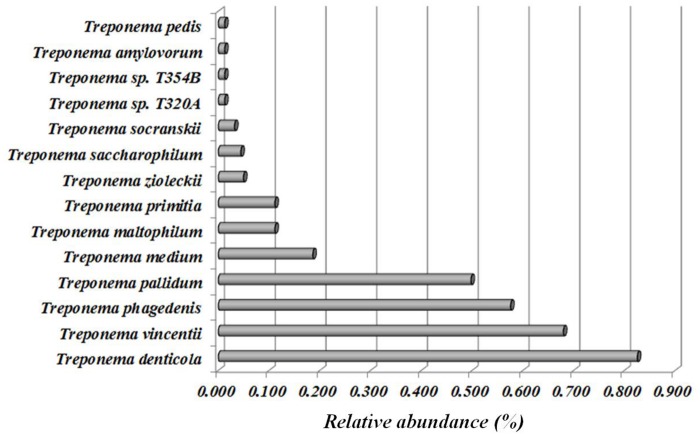
Relative abundance of *Treponema* species in rumen samples from 8 lactating dairy cows.

The relative abundance of the most common *Treponema* spp. present in fecal samples of lactating dairy cows housed with the cows diagnosed with DD are illustrated in Fig. 11. All of the six *Treponema* spp. that were highly associated with active DD lesions were present in fecal samples (Fig. 11). We also evaluated the relative abundance of *Treponema* spp. in rumen fluid samples of lactating dairy cows housed with the cows diagnosed with DD (Fig. 12). Four of the six *Treponema* spp. that were highly associated with active DD lesions were present in rumen fluid samples (Fig. 12).

## Discussion

One of the major goals of microbiome research is to describe the structure and function of microbiomes in health and active disease states [38]. The present study identified shifts in the microbiome of cows diagnosed with DD lesions when compared with healthy skin, and bolstered the concept that DD is a polymicrobial disease, with *Treponema* spp. likely playing a critical role in disease pathogenesis. Additionally, we found the same subtypes of *Treponema* spp. in fecal and rumen samples, pointing to the gut microbiome as an important reservoir for microbes related to DD pathogenesis. We used a combination of two classifications to record disease stages of DD in dairy cow hind feet [25],[29]. However, when we considered the clinical characteristics of DD in the context of the clustering of microbiomes observed in the present study and to facilitate analysis and interpretation of the data, we decided to use a simpler terminology to characterize the lesions stages of DD. Ulcerative lesions of DD are usually painful, associated with lameness, and require individual antimicrobial treatment [39]. In the present study, cows exhibiting stages M1, M2 and M4.1 (lesion stages with an ulcerative area) all had similar microbiomes; therefore, we propose to name the lesions with an ulcerative area as ‘active lesions’. The other DD lesion types (M3 and M4) are typically characterized by a firm scab, hyperkeratosis, proliferative overgrowth, absence of an ulcerative area, absence of pain, and are not associated with lameness. Additionally, it was also observed in the present study, that the microbiome of the non-ulcerative lesion stages (M3 and M4) were clearly distinct from the ulcerative lesion states (‘active lesion’, M1, M2 and M4.1) and from healthy skin. Therefore, we propose to name these non-ulcerative lesions as ‘inactive lesions’. A recent reclassification of DD lesions into 6 different stages (Iowa DD lesion scoring system) by Krull et al. (2014) [15] also aimed to accommodate microbiome results of increased presence of *Treponema* spp. and morphological changes occurring during disease progression. Although, the results presented here resemble the findings of Krull et al. (2014) [15] in many respects, with both studies observing increases of *Treponema* spp. in ulcerative lesions, we believe that our binary classification of DD lesions into ‘active’ or ‘inactive’ is a more useful scoring system for microbiome studies because it simplifies the characterization of DD lesions by integrating the most clinically relevant aspects of the lesions and emphasizes the distinction between their microbiomes.

An additional factor considered in the present study was the comparison of samples from superficial (the first 2 mm) and deep strata (i.e., the remainder of the sample of healthy skin or DD lesion containing dermis, epidermis and hypodermis). Previous studies using FISH revealed that *Treponema* spp. are seen mostly in the deeper parts of DD lesions, near the interface with healthy tissue [11],[19]. Furthermore, an earlier study from our research team, which used cloned 16S rRNA genes and Sanger-sequencing technology to characterize the microbiomes of different strata of DD lesions, revealed an increased presence of spirochetes in the deep stratum compared to both superficial and intermediate strata [14]. However, in the present study, the differences in microbiomes between the superficial and deep strata were found to be minor. In healthy skin, the Chao1 diversity index in the deep stratum was lower than that of the superficial stratum, whereas in active DD lesions, the Chao1 diversity index in deep stratum was higher than that of the superficial stratum, suggesting that conditions deeper into active DD lesions may favor survival of a greater diversity of microbes. A second, minor difference between strata occurred in active DD lesions, in which the bacterium *Candidatus Aemobophilus asiaticus* had greater relative abundance in the superficial stratum than in the deep stratum. On the other hand, when healthy skin, active DD lesions, and inactive lesions were analyzed together, no significant differences at the phylum level or in the discriminant and screening analyses were detected for superficial and deep strata. When compared with the results of Santos et al. (2012) [14], the current study used a much more comprehensive approach, including larger sample sizes, different types of lesions and advanced high-throughput technology for 16S rRNA gene sequencing (Illumina MiSeq platform). Taken together, these methodological differences might explain the differences between the two studies, suggesting that studies combining high-throughput sequencing, large sample sizes and different lesion types yield a deeper and more accurate discovery of microbial species, and consequently the strata of the samples become a less critical factor in determining microbiome structural shifts in healthy skin and DD lesions. *Candidatus Amoebophilus asiaticus* was the only bacterium with different relative abundance between deep and superficial strata.

The structure of the foot skin microbiome at the phylum level described in the present study resembles in some respects the structures reported in previous studies of microbial diversity in healthy skin and DD lesions [13],[14],[15]. As discussed above, our research group previously used a culture-independent method and Sanger sequencing of cloned 16S rRNA genes to characterize the microbiomes of different strata of DD lesions [14], showing that the deep stratum was dominated by the phyla Spirochaetae (46%), Firmicutes (35%), Bacteroidetes (7%), Tenericutes (6%) and Proteobacteria (5.5%). Interestingly, those results are very similar to the phylum structure of active DD lesions described in the present study: Spirochetes (43.5%), Firmicutes (20.5%), Bacteroidetes (15.9%), Tenericutes (6.4%) and Proteobacteria (7.2%). However, the phylum distribution of inactive DD lesions was noticeably different between the two studies from our group; in the present study it was dominated by Firmicutes (39.6%) and Bacteroidetes (20.9%), followed by Proteobacteria (13.1%), Spirochetes (9%) and Actinobacteria (2.7%). The phylum distribution in the present healthy samples was dominated by Firmicutes and Actinobacteria, two phyla highly associated with healthy skin in previous studies [13],[14],[15]. These results suggest that the microbiome structure at the phylum level shifts considerably between healthy skin and DD lesions and supports the theory that healthy foot skin is dominated by Firmicutes, whereas Spirochetes are the most prevalent phylum in DD lesions. Furthermore, these results indicate that the microbiome of inactive lesions is more similar to that of healthy skin than to that of active DD lesions, at least at the phylum level.

The next step we took to differentiate healthy skin and DD lesions was a discriminant analysis that identifies and ranks microbes important for microbiome distinction based on species-level relative abundance to create canonical scores that allow separation of clusters representing healthy skin and DD lesions. Some of top-ranked bacteria in DD lesions included a variety of spirochetes—*T*. *denticola*, *T*. *maltophilum*, *T*. *paraluiscuniculi*, *T*. *phagedenis*—and a group of other bacteria not previously associated with DD lesions: *Candidatus Amoebophilus asiaticus*, *Telmatospirillum siberiense*, *Porphyromonas canis*, and *Alkaliphilus crotonatoxidans*. Likewise, certain microbes (*Bacteroides denticanum*, Oscillospiraceae and *Arcanobacterium bernardidae*) were more prevalent in healthy skin and important for separating the microbiome of healthy skin from that of DD lesions. Some major bacteria more prevalent in active DD lesions than in inactive DD lesions included a series of Spirochetes (*T*. *denticola*, *T*. *maltophilum*, *T*. *paraluiscuniculi*, *T*. *phagedenis* and *Treponema putidum*), *Bacteroides denticum*, *Porphyromonas asaccharolytica*, *Acholeplasma palmae*, *Soehngenia saccharlytica*, *Blautia hansenni* and *Dichelobacter nodosus*, a bacterium previously associated with DD [19]. In contrast, the following species were more prevalent in inactive DD lesions than in active ones: *Mehiotermus granaticus*, *Sedimentibacter hidroxybenzoicus*, *Treponema calligyrum*, *Porphyromonas canis*, *Campylobacter curvus*, *Clostriudium thermoalcaliphilum*, *Psychroflexus gondwanensis* and *Candidatus Amoebophilus asiaticus*.

To confirm and refine the results of the discriminant analysis, we next performed a screening analysis using the false discovery rate and correcting for multiple comparisons. The screening analysis underscored the critical importance of *Treponema* spp. for differentiating the microbiomes of healthy skin and inactive DD lesions from that of active DD lesions. The *Treponema* spp. that were greatly increased in active DD lesions compared to healthy skin and even inactive lesions included *T*. *denticola*, *T*. *medium*, *T*. *maltophilum*, *T*. *paraluiscuniculi*, *T*. *phagedenis*, *T*. *putidum* and *T*. *vincentii*. Detecting this group of *Treponema* spp. confirmed the findings of several earlier studies [13],[14],[16],[28],[40], and also revealed the importance of *T*. *paraluiscuniculi* in DD lesions, a species previously considered unimportant for this disease [41]. *T*. *paraluiscuniculi* is known as an etiological agent of venereal syphilis in rabbits, but it is not related to syphilis in humans. The massive increase in *Treponema* spp. found in the present study between healthy foot skin and DD lesions was also observed by Krull et al. (2014) [15], in which the Spirochaetaceae family increased from 0.0% in healthy foot skin to 94.3% in DD lesions. The majority of these *Treponema* spp. had already been associated with bovine DD in multiple studies[13],[14], [40]. Evans et al. (2008) [16] reported that *T*. *phagedenis-like*, *T*. *medium/T*. *vincentii-like* and *T*. *putidum/T*. *denticola-like* were present in bovine DD samples in abundances of 98%, 96.1% and 74.5%, respectively. These results are in line with the findings of the present study, which also identified these *Treponema spp*. to be among the most important types in active DD lesions, and supporting their likely critical role in the development of DD lesions.

A novel bacterium that also seems important for DD lesions is *Candidatus Amoebophilus asiaticus*, whose presence was markedly increased in both active and inactive DD lesions compared with healthy skin samples. *Candidatus Aemobophilus asiaticus* is a gram negative bacterium that belongs to the phylum Bacteroidetes and it has been reported to be an obligate intracellular symbiont of amoebas [42],[43]. Amoebas play a host role for many intracellular bacteria and they are important predators of prokaryotic and eukaryotic microorganisms, controlling environmental microbial communities and also serving as vectors and reservoirs of human disease agents such as *Legionella pneumophila* and *Mycobacterium avium* [44],[45]. The high relative abundance of *Candidatus Amoebophilus asiaticus* encountered in active and inactive DD lesions is suggestive that this bacterium might play an active role in the pathogenesis of DD. However, a second possibility is that this bacterium is merely an indirect indicator of the presence of amoeba (its natural host) in DD lesions without being necessarily involved in DD pathogenesis. Amoebas are natural predators of bacteria, and DD lesions are heavily contaminated with bacteria, hence the presence of amoebas and consequently of its symbiont *Candidatus Aemobophilus asiaticus*.

Another bacterium considered in previous studies to be a major player in the pathogenesis and polymicrobial character of DD lesions is *Dichelobacter nodosus* [19],[46]. However, we did not detect an increase in *Dichelobacter nodosus* abundance in active DD lesions versus inactive DD lesions or healthy skin samples, challenging the importance of this species in DD pathogenesis. Similarly, Krull et al. (2014) [15] found no difference in *Dichelobacter nodosus* between DD lesions and healthy skin.

In the present study, the markers of the microbiome of healthy skin were diverse, but dominated by bacteria belonging to the phyla Firmicutes and Actinobacteria, which is in line with the findings of several studies for healthy foot skin [13],[14],[15]. Diversity was greatest in healthy skin samples, and became gradually lower in inactive DD lesions and active DD lesions, also in agreement with the findings of Krull et al. (2014) [15]. Interestingly, the increase of *Treponema* spp. in inactive lesions when compared to healthy skin was minor, with only *T*. *phagedenis-like* having more than 1% relative abundance in inactive DD lesions, suggesting that *Treponema spp*. do not play an important role in inactive stages of the disease. In fact, the microbial markers of inactive DD lesions were *Porphyromonas* spp., *Alkaliphilus crotonatoxidans*, *Sedimentibacter hydroxybenzoicus*, and *Filifactor villosus*, which exhibited marked increases when compared to healthy skin and active DD lesions, illustrating the distinct structure of the microbial community dominating in inactive DD lesions.

Our results support the concept that *Treponema spp*. are the most important group involved in DD pathogenesis. A diversity of *Treponema spp*. had previously been described in DD lesions, suggesting a “polytreponemal” etiology for the disease [12],[47]. Many facts in the literature support this theory, including a study by Gomez et al. (2012) [23] that attempted to induce the disease by inoculating skin with a pure culture of a single *Treponema* species, but failed to cause disease in the majority of animals inoculated. In the present study, we investigated how the most important *Treponema* spp. found in active DD lesions were correlated among themselves. The linear correlation matrix for *T*. *denticola*, *T*. *maltophilum*, *T*. *medium T*. *putidum*, *T*. *phagedenis and T*. *paraluiscuniculi* shows a high degree of correlation between the *Treponema* spp. associated with DD, supporting the concept that DD etiology is indeed “polytreponemal”.

The reservoir for the pathogens associated with bovine DD remains unclear. We sought in the present study to test the possibility that bovine gut microbes are a potential reservoir for the pathogens related to DD lesions. Thus, we chose a group of cows from the same farm on which we sampled most of the DD lesions, and investigated whether the microbiomes of feces and rumen contained the same *Treponema* spp. that were found to be highly associated with DD lesions. As we expected, the bacteria associated with active DD lesions were nearly ubiquitously present in the sampled fecal and ruminal microbial communities, suggesting that gut microbes in dairy cows might be the reservoir for pathogens causing DD lesions. To our knowledge, this is the first scientific report to demonstrate the presence of DD-causing *Treponema* spp. in the rumen and feces of lactating cows. However, *T*. *denticola*, *T*. *pedis*, and *T*. *phagedenis* were previously identified in both the dairy environment and in DD lesions using high-throughput sequencing [28]. In summary, the results of the present study suggest that the rumen and distal gastrointestinal tract of dairy cows harbor the microbes associated with DD pathogenesis. Targeting the gut to decrease DD pathogen pressure in the environment is a logical potential disease control strategy and warrants further investigation.

## Conclusions

Our data support the concept that DD is a polymicrobial disease, with active DD lesions having a markedly distinct microbiome dominated by *T*. *denticola*, *T*. *maltophilum*, *T*. *medium*, *T*. *putidum*, *T*. *phagedenis* and *T*. *paraluiscuniculi*. All but one of these species were found in rumen and fecal microbiomes, suggesting that the gut microbiota might be a reservoir for microbes involved in DD pathogenesis. Further investigation into the potential role of the gut microbiome as a reservoir for pathogens leading to DD development and of prophylactic measures to control the potential environmental shedding of these pathogens is needed. Additionally, the bacterium *Candidatus Amoebophilus asiaticus* was highly abundant in active and inactive DD lesions.

## Supporting Information

S1 Fig Canonical scores 1 for bacterial taxa that were found to be significant for the discriminant analysis displayed in Fig. 4. (TIF)

S2 Fig Canonical scores 2 for bacterial taxa that were found to be significant for the discriminant analysis displayed in Fig. 4. (TIF)

S3 Fig Canonical scores 3 for bacterial taxa that were found to be significant for the discriminant analysis displayed in Fig. 4. (TIF)

S4 Fig Discriminant analysis of healthy skin samples and lesion stages M1, M2, M3, M4 and M4.1 of digital dermatitis. (TIF)

S5 Fig Percentage increase of bacterial types from active digital dermatitis lesions to healthy skin. The Y axis represents the robust LogWorth of the false discovery rate and the X axis represents the percentage increase in relative abundance when comparing active digital dermatitis lesions to healthy skin. The sizes of the circles represent the effect size and the colors represent the relative abundance of each individual bacterial type in healthy skin (color legend upper right corner). Green line represents *P* < 0.00005.
(TIF)

S6 Fig Percentage increase of bacterial species from active digital dermatitis lesions to inactive digital dermatitis lesions. The Y axis represents the robust LogWorth of the false discovery rate and the X axis represents the percentage increase in relative abundance when comparing healthy skin samples to active digital dermatitis lesions. The sizes of the circles represent the effect size and the colors represent the relative abundance of each individual bacterial type in inactive digital dermatitis lesions (color legend upper right corner). Green line represents *P* < 0.00005.
(TIF)

S7 Fig Percentage increase of bacterial species from inactive digital dermatitis lesions to healthy skin. The Y axis represents the robust LogWorth of the false discovery rate and the X axis represents the percentage increase in relative abundance when comparing healthy skin samples to active digital dermatitis lesions. The sizes of the circles represent the effect size and the colors represent the relative abundance of each individual bacterial type in healthy skin (color legend upper right corner). Green line represents *P* < 0.00005.
(TIF)

S8 Fig Relative abundance of the major bacterial species associated with digital dermatitis (DD) in healthy skin, lesion stages M2, M3, M4, and M4.1. Bacterial types were selected based on top-ranked robust LogWorth of the false discovery rate and average relative abundance of bacterial types in healthy skin, inactive DD lesions, and active DD lesions. Asterisks mean significance. *P < 0.05.
(TIF)

S1 Table Canonical scores 1, 2 and 3 for bacterial taxa that were found to be significant for the discriminant analysis displayed in S4 Fig. (PDF)
