# Supplementary figures and images for: Altered Microbiomes in Bovine Digital Dermatitis Lesions, and the Gut as a Pathogen Reservoir
Source: PLoS One. 2015 Mar 17;10(3):e0120504. doi: 10.1371/journal.pone.0120504 (PMC4362943; doi:10.1371/journal.pone.0120504)

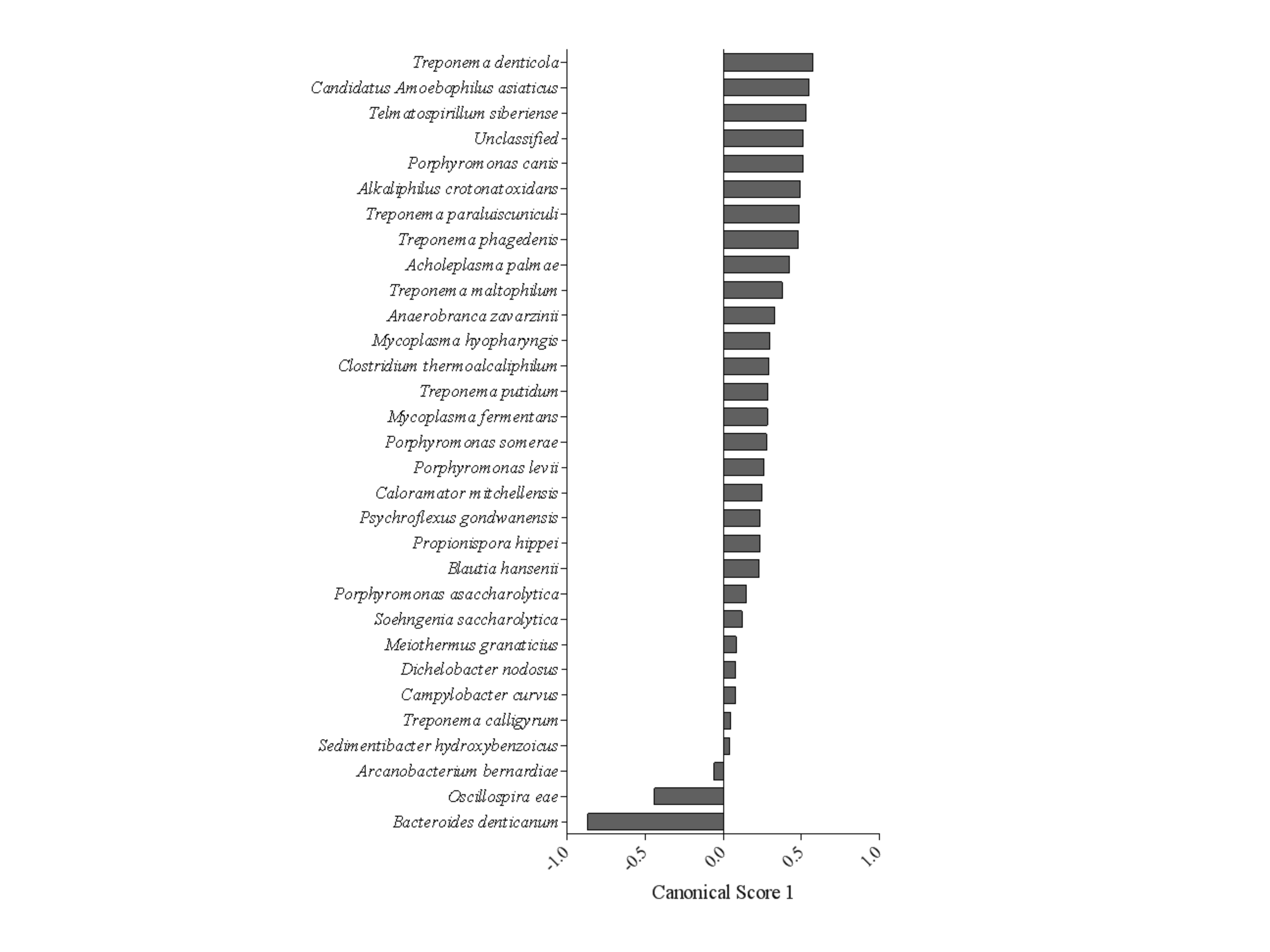

Supplement: S1 Fig — (TIF) [file pone.0120504.s001.tif]

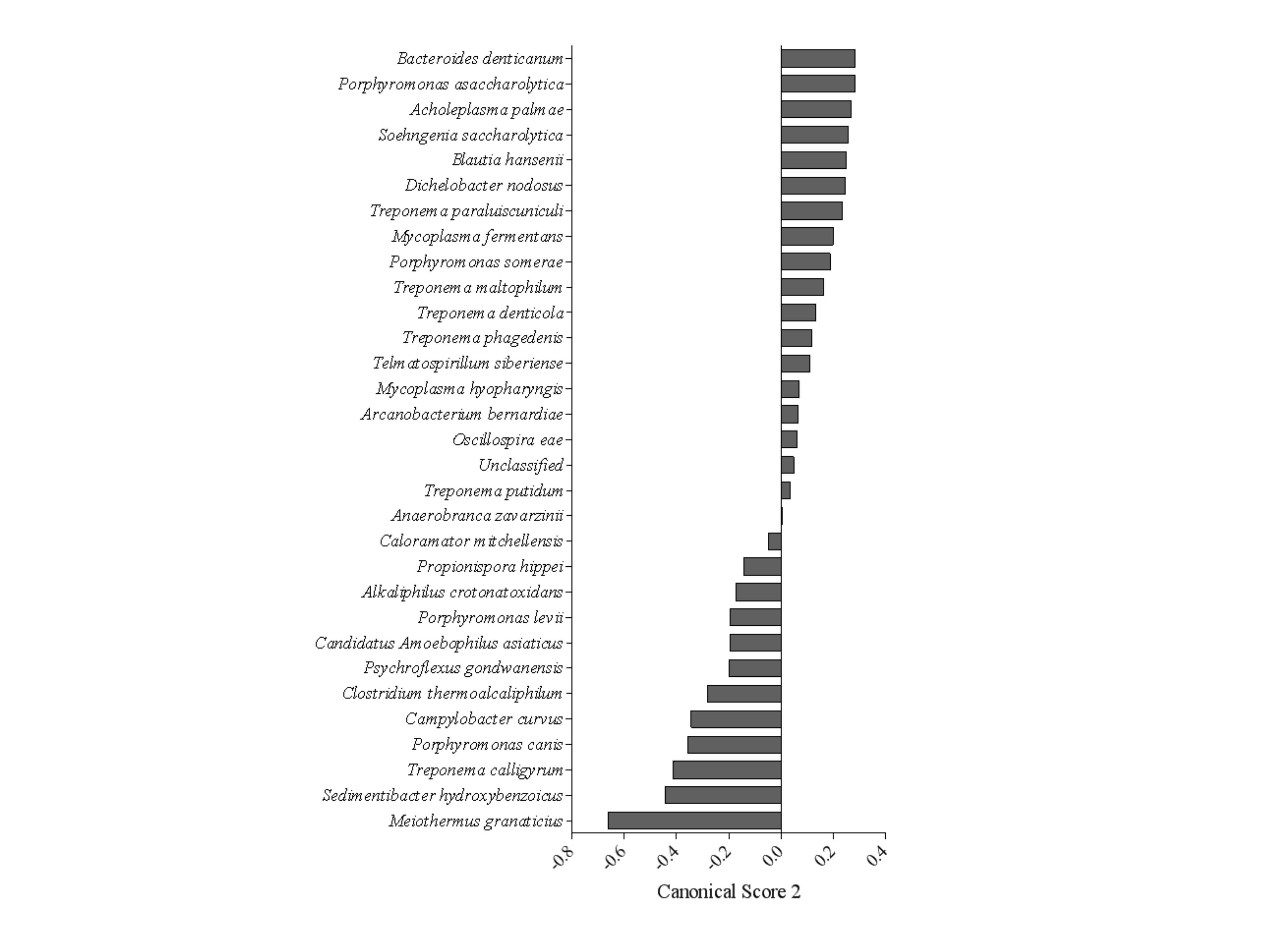

Supplement: S2 Fig — (TIF) [file pone.0120504.s002.tif]

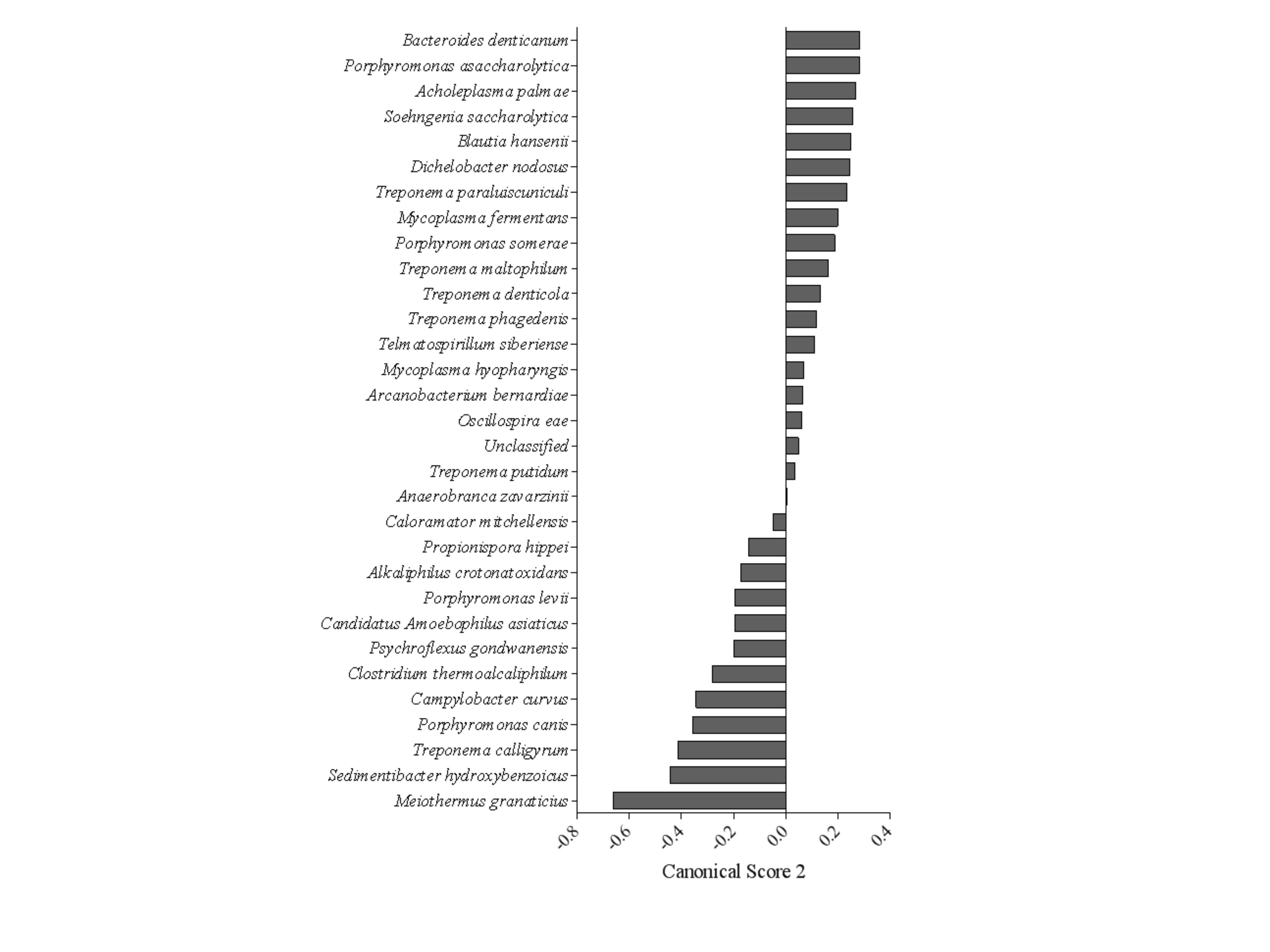

Supplement: S3 Fig — (TIF) [file pone.0120504.s003.tif]

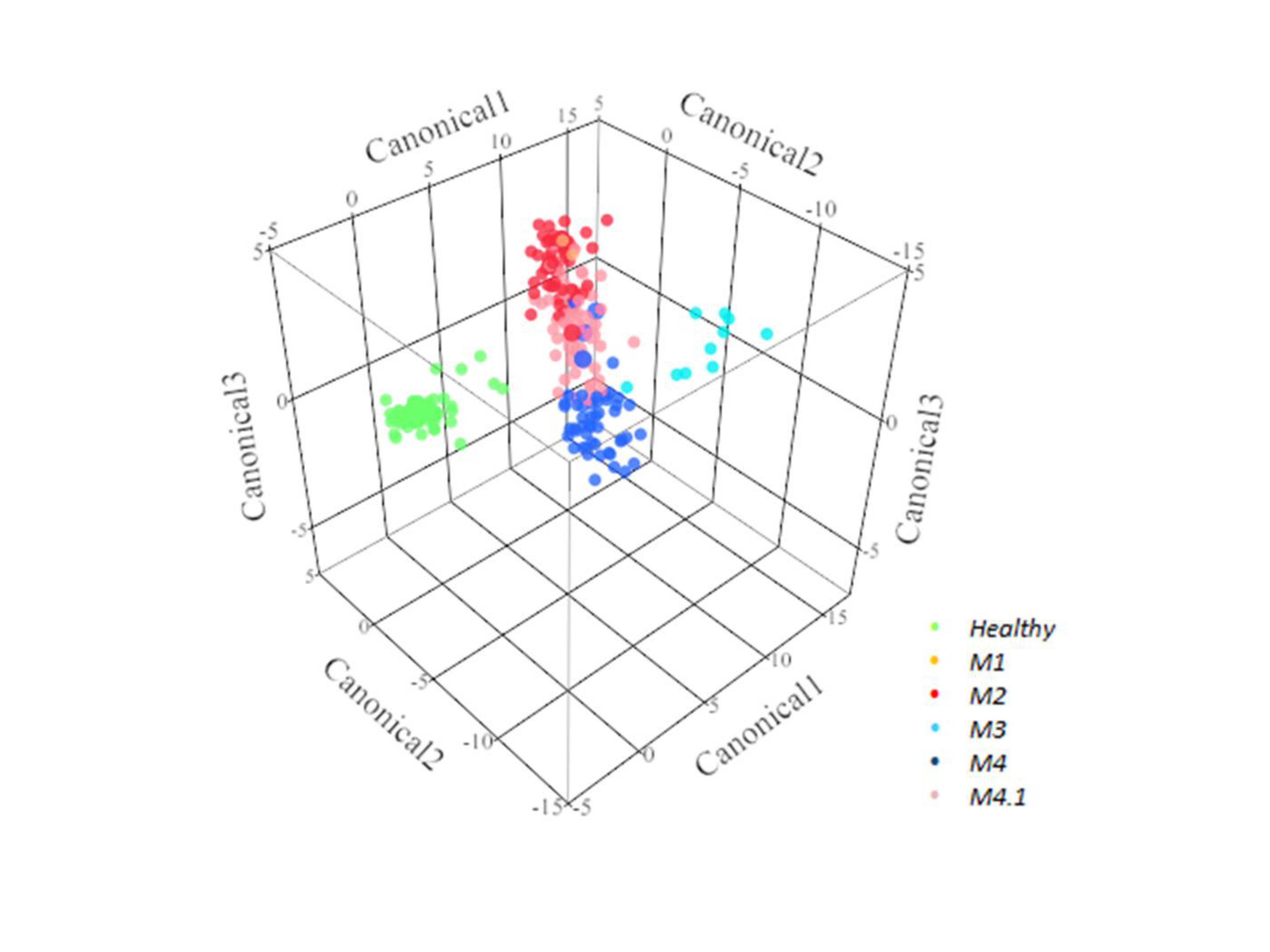

Supplement: S4 Fig — (TIF) [file pone.0120504.s004.tif]

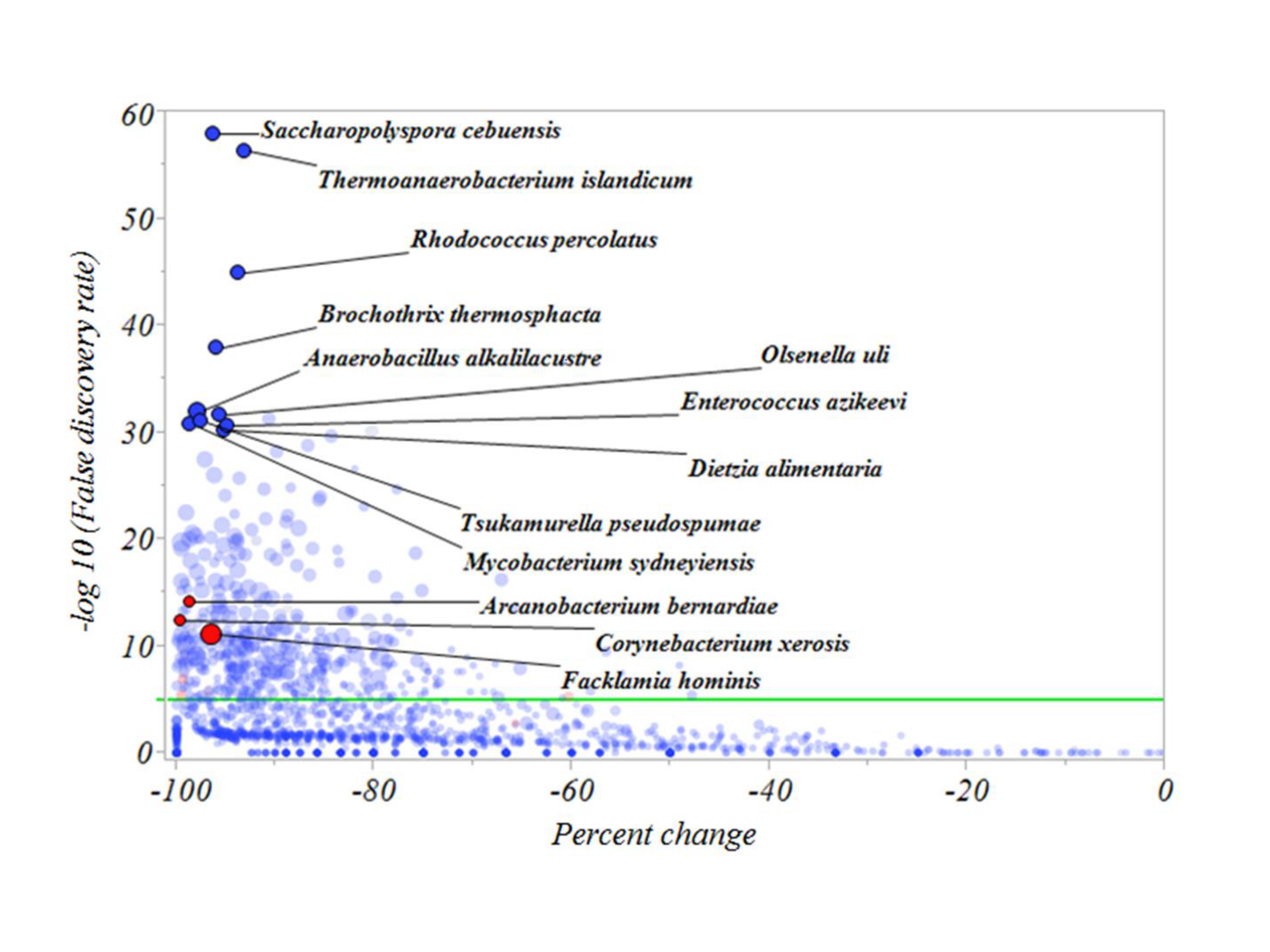

Supplement: S5 Fig — The Y axis represents the robust LogWorth of the false discovery rate and the X axis represents the percentage increase in relative abundance when comparing active digital dermatitis lesions to healthy skin. The sizes of the circles represent the effect size and the colors represent the relative abundance of each individual bacterial type in healthy skin (color legend upper right corner). Green line represents P < 0.00005. (TIF) [file pone.0120504.s005.tif]

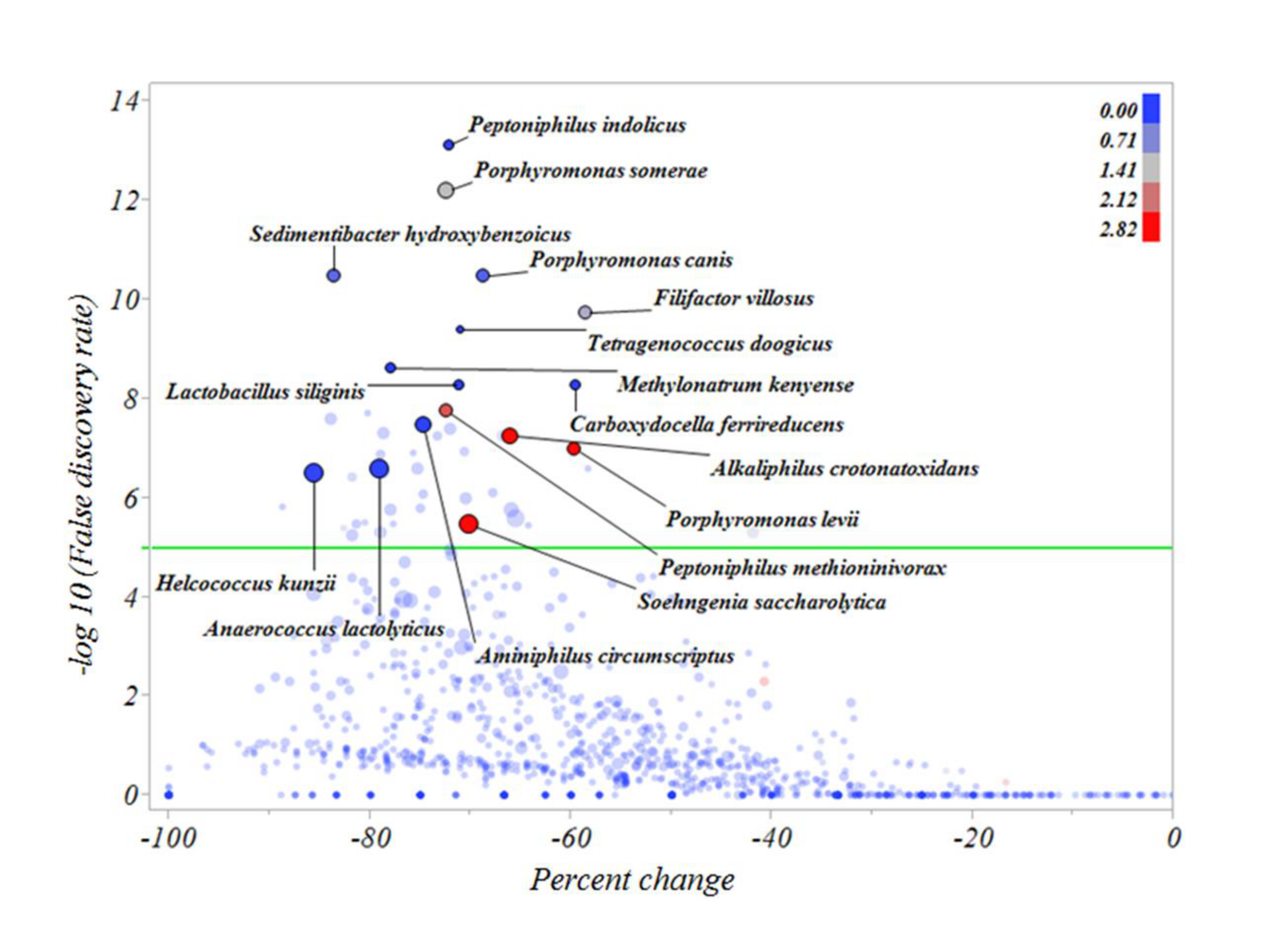

Supplement: S6 Fig — The Y axis represents the robust LogWorth of the false discovery rate and the X axis represents the percentage increase in relative abundance when comparing healthy skin samples to active digital dermatitis lesions. The sizes of the circles represent the effect size and the colors represent the relative abundance of each individual bacterial type in inactive digital dermatitis lesions (color legend upper right corner). Green line represents P < 0.00005. (TIF) [file pone.0120504.s006.tif]

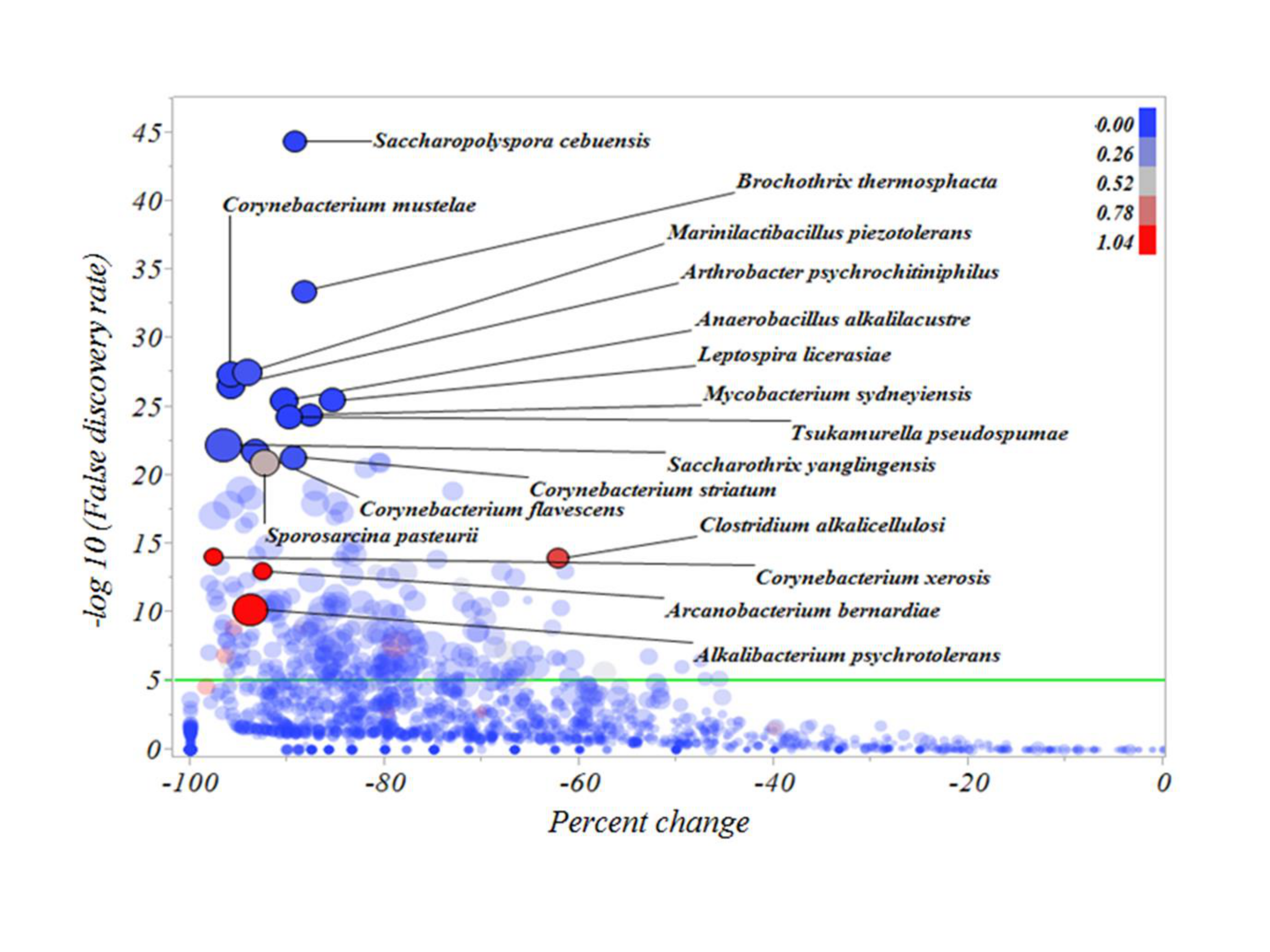

Supplement: S7 Fig — The Y axis represents the robust LogWorth of the false discovery rate and the X axis represents the percentage increase in relative abundance when comparing healthy skin samples to active digital dermatitis lesions. The sizes of the circles represent the effect size and the colors represent the relative abundance of each individual bacterial type in healthy skin (color legend upper right corner). Green line represents P < 0.00005. (TIF) [file pone.0120504.s007.tif]

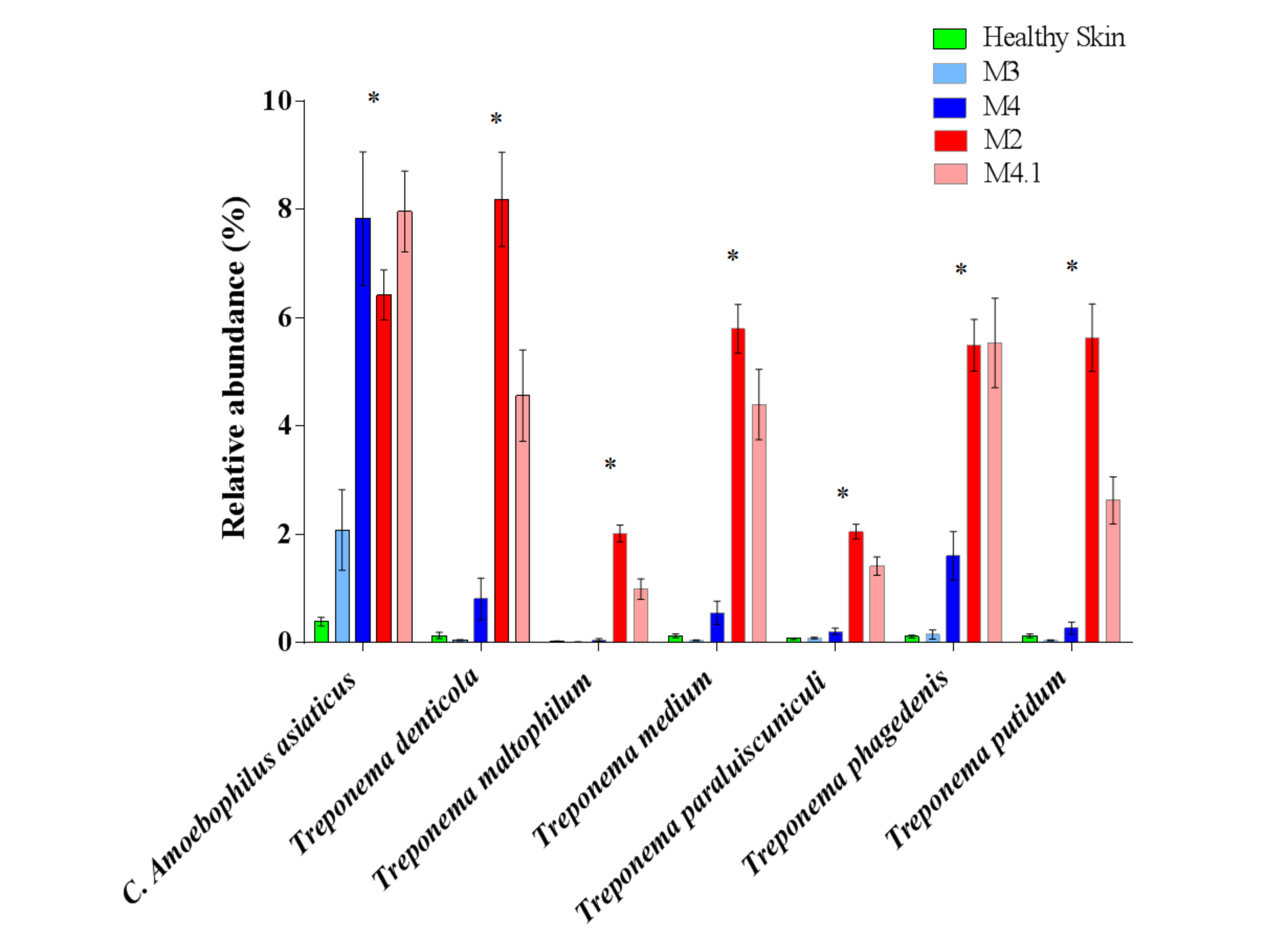

Supplement: S8 Fig — Bacterial types were selected based on top-ranked robust LogWorth of the false discovery rate and average relative abundance of bacterial types in healthy skin, inactive DD lesions, and active DD lesions. Asterisks mean significance. *P < 0.05. (TIF) [file pone.0120504.s008.tif]
