# Supplementary material for: Altered Microbiomes in Bovine Digital Dermatitis Lesions, and the Gut as a Pathogen Reservoir
Source: PLoS One. 2015 Mar 17;10(3):e0120504. doi: 10.1371/journal.pone.0120504 (PMC4362943; doi:10.1371/journal.pone.0120504)
Supplement: S1 Table — (PDF) [file pone.0120504.s009.pdf]

Canonical 1		Canonical 2		Canonical 2	
**Bacterial species**	**Score**	**Bacterial species**	**Score**	**Bacterial species**	**Score**
Treponema paraluiscuniculi	0.95	***Porphyromonas asaccharolytica***	1.32	***Candidatus Phytoplasma brasiliense***	0.65
Anaerococcus prevotii	0.94	***Arcobacter marinus***	0.92	***Finegoldia magna***	0.60
Candidatus Phytoplasma prunorum	0.89	***Candidatus Phytoplasma brasiliense***	0.48	***Meiothermus granaticius***	0.58
Clostridium thermoalcaliphilum	0.78	***Hydrocarboniphaga daqingensis***	0.48	***Helcococcus sueciensis***	0.49
Treponema denticola	0.59	***Alkaliphilus crotonatoxidans***	0.45	***Peptostreptococcus anaerobius***	0.37
Candidatus Amoebophilus asiaticus	0.57	***Oligella ureolytica***	0.35	***Prevotella dentasini***	0.36
Telmatospirillum siberiense	0.55	***Anaerococcus prevotii***	0.33	***Gemella cunicula***	0.29
Treponema phagedenis	0.54	***Tepidimicrobium ferriphilum***	0.30	***Treponema paraluiscuniculi***	0.27
Acholeplasma palmae	0.53	***Acholeplasma palmae***	0.28	***Tepidimicrobium ferriphilum***	0.25
Soehngenia saccharolytica	0.46	***Mogibacterium timidum***	0.24	***Anaerococcus prevotii***	0.24
Arcobacter marinus	0.45	***Catonella morbi***	0.22	***Selenomonas artemidis***	0.22
Alkaliphilus crotonatoxidans	0.45	***Treponema phagedenis***	0.20	***Soehngenia saccharolytica***	0.21
Corynebacterium propinquum	0.41	***Treponema paraluiscuniculi***	0.19	***Campylobacter ureolyticus***	0.21
Johnsonella ignava	0.39	***Bacteroides rodentium***	0.18	***Escherichia coli***	0.20
Porphyromonas cansulci	0.38	***Treponema putidum***	0.17	***Treponema maltophilum***	0.16
Treponema medium	0.37	***Brochothrix thermosphacta***	0.17	***Mobiluncus mulieris***	0.14
Psychroflexus gondwanensis	0.36	***Mycoplasma fermentans***	0.11	***Bacteroides rodentium***	0.11
Treponema putidum	0.35	***Candidatus Phytoplasma phoenicium***	0.10	***Treponema putidum***	0.10
Succiniclasticum ruminis	0.34	***Pseudomonas azotoformans***	0.10	***Oligella ureolytica***	0.09
Mycoplasma fermentans	0.34	***Treponema maltophilum***	0.09	***Candidatus Phytoplasma phoenicium***	0.08
Bacteroides graminisolvens	0.34	***Johnsonella ignava***	0.08	***Treponema phagedenis***	0.08
Oligella ureolytica	0.32	***Treponema medium***	0.08	***Hydrocarboniphaga daqingensis***	0.05
Treponema maltophilum	0.28	***Campylobacter gracilis***	0.08	***Clostridium thermoalcaliphilum***	0.05
Treponema calligyrum	0.26	***Soehngenia saccharolytica***	0.06	***Propionispora hippei***	0.02
Porphyromonas asaccharolytica	0.24	***Escherichia coli***	0.05	***Treponema denticola***	0.02
Mogibacterium timidum	0.23	***Anaerococcus tetradius***	0.04	***Corynebacterium propinquum***	0.01
Campylobacter curvus	0.23	***Saccharothrix yanglingensis***	0.04	***Porphyromonas asaccharolytica***	0.01
Candidatus Phytoplasma	0.21	***Mobiluncus mulieris***	0.01	***Brochothrix***	0.00

fragariae				***thermosphacta***	
Tepidimicrobium ferriphilum	0.20	***Propionispora hippei***	-0.01	***Porphyromonas cansulci***	-0.04
Pelotomaculum isophthalicicum	0.19	***Telmatospirillum siberiense***	-0.03	***Candidatus Phytoplasma fragariae***	-0.05
Selenomonas artemidis	0.18	***Prevotella dentasini***	-0.04	***Mycoplasma fermentans***	-0.05
Propionispora hippei	0.18	***Campylobacter curvus***	-0.06	***Saccharothrix yanglingensis***	-0.05
Sedimentibacter hydroxybenzoicus	0.16	***Treponema denticola***	-0.06	***Anaerovibrio lipolyticus***	-0.08
Meiothermus granaticius	0.14	***Anaerovibrio lipolyticus***	-0.07	***Acholeplasma palmae***	-0.09
Campylobacter gracilis	0.12	***Psychroflexus gondwanensis***	-0.07	***Psychroflexus gondwanensis***	-0.10
Mobiluncus mulieris	0.10	***Selenomonas artemidis***	-0.11	***Treponema calligyrum***	-0.10
Escherichia coli	0.10	***Sedimentibacter hydroxybenzoicus***	-0.12	***Catonella morbi***	-0.10
Anaerococcus tetradius	0.09	***Desulfovibrio simplex***	-0.17	***Mogibacterium timidum***	-0.12
Campylobacter ureolyticus	0.08	***Bacteroides graminisolvens***	-0.17	***Telmatospirillum siberiense***	-0.12
Anaerovibrio lipolyticus	0.08	***Clostridium thermoalcaliphilum***	-0.20	***Campylobacter gracilis***	-0.14
Desulfovibrio simplex	0.04	***Succiniclasticum ruminis***	-0.20	***Treponema medium***	-0.16
Peptostreptococcus anaerobius	0.04	***Candidatus Phytoplasma fragariae***	-0.22	***Pseudomonas azotoformans***	-0.17
Catonella morbi	-0.01	***Corynebacterium propinquum***	-0.22	***Desulfovibrio simplex***	-0.17
Bacteroides rodentium	-0.04	***Pelotomaculum isophthalicicum***	-0.23	***Bacteroides graminisolvens***	-0.21
Saccharothrix yanglingensis	-0.08	***Candidatus Amoebophilus asiaticus***	-0.24	***Candidatus Amoebophilus asiaticus***	-0.25
Prevotella dentasini	-0.23	***Treponema calligyrum***	-0.29	***Pelotomaculum isophthalicicum***	-0.25
Pseudomonas azotoformans	-0.26	***Campylobacter ureolyticus***	-0.33	***Alkaliphilus crotonatoxidans***	-0.27
Finegoldia magna	-0.30	***Porphyromonas cansulci***	-0.45	***Candidatus Phytoplasma prunorum***	-0.28
Hydrocarboniphaga daqingensis	-0.30	***Helcococcus sueciensis***	-0.46	***Johnsonella ignava***	-0.29
Candidatus Phytoplasma phoenicium	-0.31	***Candidatus Phytoplasma prunorum***	-0.60	***Sedimentibacter hydroxybenzoicus***	-0.31
Brochothrix thermosphacta	-0.37	***Finegoldia magna***	-0.81	***Campylobacter curvus***	-0.35
Helcococcus sueciensis	-0.38	***Gemella cunicula***	-0.82	***Succiniclasticum ruminis***	-0.40
Gemella cunicula	-0.42	***Peptostreptococcus anaerobius***	-0.82	***Arcobacter marinus***	-0.66
Candidatus Phytoplasma brasiliense	-0.84	***Meiothermus granaticius***	-1.88	***Anaerococcus tetradius***	-0.90
Treponema paraluiscuniculi	0.95	***Porphyromonas asaccharolytica***	1.32	***Candidatus Phytoplasma brasiliense***	0.65
